# On-site Rapid Diagnosis of Intracranial Hematoma using Portable Multi-slice Microwave Imaging System

**DOI:** 10.1038/srep37620

**Published:** 2016-11-29

**Authors:** Ahmed Toaha Mobashsher, A. M. Abbosh

**Affiliations:** 1 School of ITEE, The University of Queensland, St Lucia, 4072, Brisbane, Australia

## Abstract

Rapid, on-the-spot diagnostic and monitoring systems are vital for the survival of patients with intracranial hematoma, as their conditions drastically deteriorate with time. To address the limited accessibility, high costs and static structure of currently used MRI and CT scanners, a portable non-invasive multi-slice microwave imaging system is presented for accurate 3D localization of hematoma inside human head. This diagnostic system provides fast data acquisition and imaging compared to the existing systems by means of a compact array of low-profile, unidirectional antennas with wideband operation. The 3D printed low-cost and portable system can be installed in an ambulance for rapid on-site diagnosis by paramedics. In this paper, the multi-slice head imaging system’s operating principle is numerically analysed and experimentally validated on realistic head phantoms. Quantitative analyses demonstrate that the multi-slice head imaging system is able to generate better quality reconstructed images providing 70% higher average signal to clutter ratio, 25% enhanced maximum signal to clutter ratio and with around 60% hematoma target localization compared to the previous head imaging systems. Nevertheless, numerical and experimental results demonstrate that previous reported 2D imaging systems are vulnerable to localization error, which is overcome in the presented multi-slice 3D imaging system. The non-ionizing system, which uses safe levels of very low microwave power, is also tested on human subjects. Results of realistic phantom and subjects demonstrate the feasibility of the system in future preclinical trials.

Intracranial hematoma (ICH) is the leading cause of permanent disability and a major cause of death worldwide. This is a type of brain injury that occurs when blood vessel bursts within the cranium (skull) region, creating a pool of hematoma inside, which eventually interrupts the regular blood supply to various brain parts and increases intracranial pressure^1^,^2^. As millions of brain cells die every second^3^,^4^ from the onset of ICH, this devastating disorder deteriorates rapidly. Therefore, on-the-spot and rapid diagnostic is the governing factor of the timely medication to ensure complete recovery of the injured patient^5^,^6^. Although some existing imaging technologies, like CT and MRI, are capable of ICH diagnosis, they are time-consuming, expensive, bulky and stationary^7^,^8^. Thus, they cannot be carried by first response paramedic teams for the diagnosis purpose or be used for bed-side monitoring to observe concurrent ICH over periods of time without moving the patient.

Among other imaging modalities, electrical impedance tomography (EIT)^9^,^10^,^11^, magnetic induction tomography (MIT)^12^,^13^ and phase-shift spectroscopy^14^,^15^ have potentially demonstrated their capabilities of being portable, fast and low-cost. But these imaging systems are tested on simpler phantom models and the prototypes are barely tested in realistic environment in presence of complex phantoms and lack human tests. Nonetheless, they suffer from poor imaging resolution (about 10% of the subject diameter for EIT^10^,^11^ and around 2.5 cm for MIT^12^). Although recently reported portable electromagnetic induction based sensing system^16^ demonstrates potential non-invasiveness and ease of operation over other sensing methods^17^,^18^, it lacks the rigorous study of physiological significance for proper understanding of the measurements. However, clinicians prefer imaging over sensing in order to detect, locate and understand the profoundness of intracranial hematoma for proper medical diagnosis^9^,^10^,^11^,^12^,^13^,^14^,^15^,^16^,^17^,^18^.

Recently, wideband microwave imaging has attracted attention from the researchers as a substitutional portable diagnostic solution owing to its non-ionising, low power, non-invasive and potentially low-cost features^19^,^20^,^21^,^22^,^23^,^24^,^25^. Microwave imaging relies on the contrast of the dielectric properties between healthy and unhealthy tissue. Although utilization of microwave frequencies was pioneered for sensing systems for anomaly detection^21^,^22^,^23^,^24^,^25^ and experimental results to identify ICH^25^ are available in the literature, the imaging of the head interior is demanded to localize ICH position inside the head^26^,^27^,^28^,^29^,^30^,^31^,^32^,^33^,^34^,^35^,^36^,^37^,^38^. Again, for rapid detection of ICH over-simplification of the electromagnetic radiation is noted for the sensing systems, e.g. assumption of pulse propagation as one- or two-dimensional^23^ rather than three-dimensional (3D) spherical manner, makes the system susceptible to errors^25^ and only imaging is reliable in this regard.

Most of the reported microwave based head imaging systems^26^,^27^,^28^,^29^,^30^,^31^ concentrated on numerical verifications or using simplified head models. Nonetheless, the reported handful head imaging prototypes rely on sensing antenna’s manual^32^,^33^ and/or mechanical^34^ rotational movements, which suffer from vibrations and increase data acquisition instabilities. Nonetheless, as the antenna need to be vertically aligned and concentric to the human head, the data acquisition is highly dependent on the experience and skill of the operator. Again, scanning the exact same slice of the head for continuous monitoring is vulnerable to errors, as the exact location of the slice is undefined. More importantly, the scanning system limits the 3D localization of ICH and suffers from lack of repeatability. At the same time, these issues can potentially jeopardise the continuous monitoring of the patient upon initial diagnosis. The reported automated head imaging prototypes^35^,^36^,^37^,^38^,^39^,^40^ use complex imaging system infrastructure involving matching liquids and relatively bulky switching network^35^,^36^,^37^,^38^,^39^ in order to improve precision and accelerate data acquisition. However, they are designed for narrow band operation and require huge time (from tens of minutes to hours^40^ depending on the required imaging accuracy) for image reconstruction, which is often shortened by using a dedicated supercomputer^35^,^38^,^39^. These limitations tend to increase the fabrication and running cost and prevent mass production.

This paper reports a wideband microwave head imaging system for fast diagnosis of ICH. The system has multi-slice scanning capability for three-dimensional (3D) localization of ICH targets. The system consists of a 3D printed versatile imaging system holding an array of light-weight sensing antenna elements, a compact transceiver to transmit and receive low-level wideband signals through the antennas, a switching network which controls the data acquisition from the antenna array, and a data storing and processing unit. The system overcomes the typical limitation of wideband head imaging systems by using a compact sensing antenna array^41^,^42^,^43^,^44^. To that end, a compact and low-profile antenna is designed with wideband operating frequencies in the low microwave band needed for head imaging. The antenna demonstrates the required directional radiation patterns with low-impulse distortion as needed to significantly improve the quality of imaging^45^. Numerical analysis of the imaging scenario by using a numerical realistic human head model is performed in both of the time and frequency domains in order to understand the performance of the multi-slice head imaging system. The prototyped system is employed on a 3D anatomically and dielectrically realistic human head phantom. An improved delay-and-sum (DAS) based back-projection algorithm with an effective head permittivity model^33^ is employed for image reconstruction. A thresholding technique is applied to normalize the reconstructed images by scanning different slices and obtain 3D localization of ICH. The radiation safety of the system is verified and the system is employed on healthy human volunteers. The reconstructed images from both of the phantom and volunteers are quantitatively and statistically analysed and the efficacy of threshold normalization for preclinical trials are investigated.

## Results

### Multi-slice wideband head imaging system

**Figure 1:**
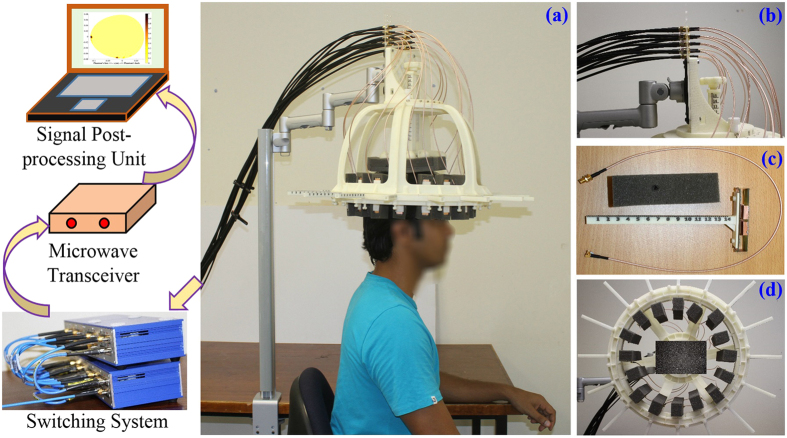
The architecture of the multi-slice microwave based head imaging system. (**a**) Proposed preclinical multi-slice wideband microwave head imaging system with the schematic representation of the interconnections. (**b**) The Photograph of the 3D printed head imaging crown mount, showing the adjustable joints, height adjuster for multi-slice scanning and cable holder. (**c**) The photograph of the scale-engraved antenna holder with the separator and utilized lightweight excitation cable. (**d**) Photograph of the 3D printed head imaging crown depicting the orientations of the sensing antenna holders and the top height adjusting support.

The architecture and different components of the proposed preclinical wideband head imaging system are illustrated in Fig. 1. The system primarily consists of an adjustable head imaging crown, an array of compact sensing antennas, an adjustable mounting stand, a switching system, a compact microwave transceiver and a signal processing and image creation unit. The different parts of the head imaging crown are fabricated using the 3D printing facility (DTM Sinterstation, 3D Systems, Inc.) of the University of Queensland, Australia and finally assembled. Acrylonitrile butadiene styrene (ABS) plastic powder, which has low dielectric constant at microwave frequencies and strong, durable finishing, is chosen for the 3D printing process. The crown is designed such that it can be mounted from the top using a suitable stand, which has the flexibility of height with both vertical and horizontal angular adjustments. A low-profile, compact unidirectional antenna is designed for the system to transmit and receive signals of wide bandwidth to and from the imaged human head. Each antenna is mounted on a 3D printed mount with two nylon screws. A foam block, which is transparent to microwave signals, is used as antenna separator from the head. A box having the footprint of the antenna’s top portion is cut and trimmed from the foam leaving a foam space of 10 *mm*, which is the constant distance between the head and antenna surface during imaging. An array of 16 antenna holders containing the compact antennas is mounted inside the crown where the arms can be extended according to the size of the head. A scale is engraved on the antenna mount in order to back-calculate the shape of the imaged head and define the external contour of the imaged head in the imaging algorithm. To facilitate multi-slice scanning of the head, a height adjuster, which touches the tip of head, and an engraved scale are used to assist in setting up an appropriate height at which a slice of the head needs to be scanned. Low-loss and lightweight cables are connected to the sensing array elements and terminated at a cable holder, which is also fixed with the mounting stand to resist any twisting and turning of antennas when used. Low-loss coaxial cables further connect the cable holder to the switching system using USB-8SPDT-A18 switches^46^. An Agilent N7081A microwave transceiver^47^ is employed to generate microwave signals, which are transmitted through the switching system to the antenna elements in a mono-static radar approach. A laptop is used as a processing and image formation unit.

### Design and development of compact wideband antenna with directional radiation patterns

The effectiveness of the head imaging system highly depends on the performance of the utilized antennas. The imaging system requires highly efficient antennas with wide bandwidth to ensure a high resolution of the reconstructed images. Since the imaging system is portable and may operate in various environments, unidirectional radiation patterns of the antennas over the entire operating bandwidth are necessary in order to overcome the environmental noise and assure that the antennas responses only to the imaged domain, the head^45^. However, the head tissues are lossy to microwave signals especially at high frequencies. Low microwave frequencies (1–2 GHz) are able to penetrate the human head due to their longer wavelengths. On the other hand, longer wavelength indicates that the required antenna would be bigger compared to those designed to operate at higher frequencies. This design requirement opposes the compact and light-weight requirement of a portable imaging system. Compactness of the sensing antennas is required to enable using more elements in the array for better imaging quality, as well as reducing the mutual-coupling effects between neighbouring antennas^30^,^31^,^32^,^33^,^34^,^35^. All the aforementioned demands of the system make the antenna design process challenging. To address these challenges, several compact antennas are proposed in the literature. For example, tapered slot or Yagi-Uda derived antennas are proposed for low microwave frequencies^31^,^48^. Although these travelling wave type antennas meet most of the requirements to radiate in low microwave frequencies, they need high profile along the direction of radiation which makes the sensing array bulky and heavy^28^, limiting its easy installation and use. For unidirectional operation, the typical monopole^43^,^44^ and patch^49^,^50^ antennas require large ground plane that increases the weight of the array and makes it unsuitable for the current application. The recently proposed folded antennas^42^ meet all the requirements, but enable using only a limited number of elements in the array owing to their high mutual-coupling which affects the impedance matching and radiation and consequently results in a degraded imaging performance. Reducing the width of the sensing antenna is one of the ways to increase the number of elements in the array; however, this procedure significantly affects the impedance matching performance of the antenna and ultimately makes it narrow/multi-band. The proposed antenna in this work demonstrates an effective way of miniaturization which enables the system to house 16 elements around the head with low mutual coupling effects.

**Figure 2:**
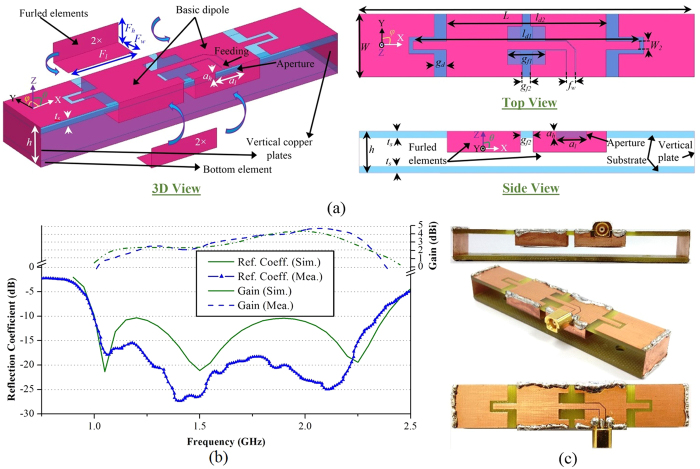
(**a**) Schematic diagrams of the designed antenna showing the 3D, top and side views and indicating the detailed dimensions of different parts. (**b**) Comparison between the measured and simulated reflection coefficient and gain versus frequency performance of the antenna over a wideband. Very low deviation in measurement from the simulated results is observed. (**c**) Different perspectives of the prototyped antenna illustrating the multiple soldering connections required for fabrication.

Figure 2(a) illustrates the geometry of the proposed compact antenna. The antenna is printed on two-slabs of low-cost glass-reinforced epoxy substrate with dielectric constant, *ε*_*r*_ = 4.4, loss tangent, tan *δ* = 0.0245 and thickness, *t*_*s*_ = 1.6 *mm*. The top and bottom slabs are connected with two vertical copper plates at the opposite sides along X-axis, creating a ring-like structure. The proposed antenna is based on a typical slot-loaded folded dipole structure^42^. The top side contains the basic dipole structure and the rest of the ring-like structure acts as a slot-loaded parasitic element. Four L-shaped copper plates are connected with the flares of the basic dipole, converting the sides of the antenna furled into its own structure. This furling technique effectively reduces the width of the folded dipole antenna and enables increasing the number of elements in the array. As the arms are furled inside the antenna, their dimensions are optimized to attain less intra-coupling effects with the top and bottom layers. It is noted that increasing or decreasing the height of the furled arm encounters intra-coupling which worsens the impedance matching of the antenna. The antenna is fed at the top layer using a co-planar waveguide feeding line. The designed antenna is prototyped and measured. The prototyped antenna is excited with Compact MCX (micro coaxial) connector at the feeding point, in order to reduce the inherent near-field radiation of the connectors.

It is noted from Fig. 2(b) that the antenna operates over the band 1–2.4 GHz, which is approximately 83% fractional bandwidth with respect to the center frequency of 1.7 GHz, assuming the −10 dB reflection coefficient as the reference. This wide bandwidth is a result of the accumulation of three different resonances. The antenna length *L* primarily determines the first resonance, while the second resonance is regulated by the longer extent, *l*_*d1*_ of the basic dipole, whereas the third resonance is mainly created by the smaller extent, *l*_*d2*_ of the basic dipole. An extended current distribution analysis indicates that the antenna operates in loop mode in the lowest resonance and in folded-dipole mode in the subsequent modes. Values of the different dimensions of the antenna mentioned in the schematic diagram are (in *mm*) : *L* = 80, *W* = 10, *h* = 10, *l*_*d1*_ = 54, *l*_*d2*_ = 38, *W*_*2*_ = 2, *a*_*h*_ = 2, *a*_*l*_ = 7.2, *F*_*h*_ = 5, *F*_*w*_ = 6, *F*_*l*_ = 17.5, *g*_*f1*_ = 9, *g*_*f2*_ = 3, *g*_*d*_ = 3, *f*_*w*_ = 2. The overall dimension of the antenna is 10 × 10 × 80 *mm*^3^, which is equivalent to 0.033 × 0.033 × 0.27 *λ*_*0*_^3^, where *λ*_*0*_ is the wavelength at lowest operating frequency (1 GHz). As demonstrated in Supplementary Table S1, the proposed antenna has much lower profile and smaller width when compared to the recently reported literature.

**Figure 3:**
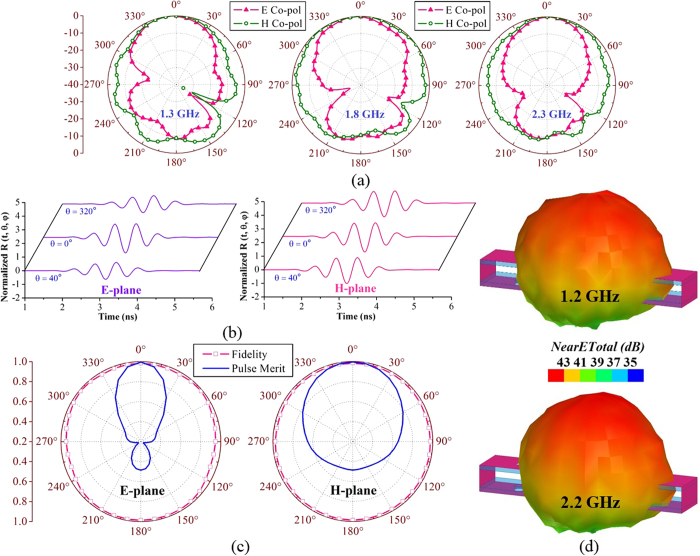
(**a**) Measured radiation patterns of the prototyped antenna at 1.3, 1.8 and 2.3 GHz in both of the E- and H-planes. (**b**) Simulated 3D radiation patterns of the antenna at 1.2 and 2.2 GHz. (**c**) The time-domain signals received by the probes placed around the antenna in different angles on both of the E- and H-planes. (**d**) The fidelity and pulse merit factor patterns of the wideband antenna in E- and H-planes resulted from the transient analysis.

The radiation performance of the antenna is also measured. It can be noted from Fig. 2(b) that the antenna attains an average gain of around 3 dBi over the operating band. The gain performance is lower at lower frequencies due to the electrically small dimensions of the antenna at those frequencies compared to higher frequencies. The radiation patterns of the antenna are measured in anechoic chamber and reported in Fig. 3(a). The antenna has directional radiation patterns in both of the E- and H-planes with an average front to back ratio of 9 dB along the direction of radiation (+ve Z-axis). The simulated 3D near-field radiation patterns at 1.2 and 2.2 GHz indicate unidirectional radiation as shown in Fig. 3(d), which assures the compatibility of the antenna in head imaging system. Again, as the imaging system is based on the time-domain algorithm, the transient characteristics of the antenna are also analysed in the near-field region, 10 cm away from the antenna surface. The details of the analysis are mentioned in the methods section. The received time-domain signals (Fig. 3(b)) illustrate that the antenna transmits visually similar wideband transient signals with gradually reduced amplitudes from *θ* = 0° in both E- and H-planes. In order to quantify the transient pulses received from the antenna, the fidelity factors^32^ and pulse merit factors^21^ of the received signals are calculated. From Fig. 3(c), it is seen that the time-domain signals radiated by the antenna have more than 90% pulse fidelities compared to the input signal at the feeding point. Thus, the antenna transmits wideband pulses with low distortion^32^. The pulse merit factor pattern, which indicates the total time-domain pulse radiation of the antenna^51^, demonstrates that the antenna is also unidirectional for transient signals in both of the E- and H-planes and suitable for systems relying on time-domain based imaging algorithms. It is noted that the phase center of the symmetrical antenna is located at the center point of the top layer and all radiations in both of the time and frequency domains at E- and H-planes start from this point. Thus, the antenna is able to scan a 2D representation^52^,^53^ of the human head located at the centerline of the antenna.

### Electromagnetic characteristics of the head when using multi-scan imaging

**Figure 4:**
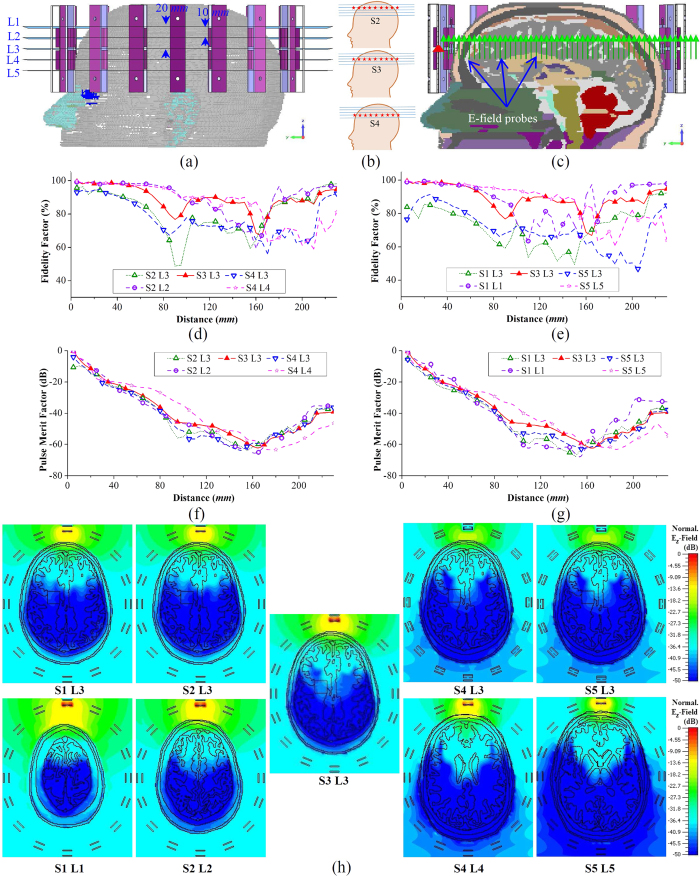
(**a**) The setup environment of the numerical analysis showing different levels (L1 to L5), while the scanning array is positioned in S3. The antennas are numbered anticlockwise starting antenna-1 in front of the forehead. (**b**) The illustration of different scanning array positions. The red stars represent the phase centres of the antennas. (**c**) The side view of the mid-sagittal plane cross section depicting the array of E-field probes utilized for the time-domain analysis. (**d**–**g**) The fidelity factor and pulse merit factor results of different time-domain pulses transmitted from antenna-1 and received at different distances inside the realistic human head model. (**h**) The total time-domain E_z_-field distributions of the horizontal cross-sections of different levels for different scanning array positions. The E_z_-fields are normalized with respect to the maximum emitted E_z_-field value from antenna-1 considering all scanning positions.

The analysis of the electromagnetic characteristics of the human head when using the proposed multi-scan head imaging system is essential to understand the system’s operation and reconstructed results. To that end, the imaging system is numerically analysed using an MRI derived human head model with actual dielectric tissue properties. As the proposed multi-scan head imaging system works on a mono-static technique, only one antenna in the array radiates and receives scattered fields at a certain time. The scanning system can scan multiple levels in a gradual manner by adjusting the top support system. As shown in Fig. 4, five levels at different vertical slices (represented with L1 to L5), which requires five different vertical array scanning positions (represented by S1 to S5) where the scanned level is located at the centerline of the antennas, are considered. However, in order to understand the electromagnetic propagations inside the head and scattering mechanism of hemorrhagic target, the electric fields in multiple levels are analysed for an individual scanning position. A hemorrhage of 2 × 2 × 0.5 cm^3^ volume is located at L3. The analyses are performed in two different sets: Firstly, three adjacent levels with 10 mm distances (L2, L3 and L4) and secondly L1, L3 and L5 (the adjacent levels with 20 mm distances among each other) are analysed to understand the relation and differences between the closely and distantly located levels.

### Time domain performance

To analyse the wave propagation inside the head, antenna-1 is excited in all 5 scanning positions (S1 to S5) with a wideband Gaussian pulse, 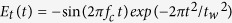, where *f*_*c*_ = 1.7 GHz is the center frequency of the operating band. As the imaging system works in mono-static approach, individual antenna analysis is performed. An array of E-field probes (Fig. 4(c)) is placed in the front side of the antenna inside the head model and the received co-polarized transient E_z_-fields are analyzed. The fidelity^32^ and pulse merit factors^51^ of the received transient signals are calculated for different scanning positions and at different levels. For the first set (L2, L3 and L4) (Fig. 4(d,f)), it is noted that the signals received for S2 L2, S3 L3 and S4 L4 yield low distortions inside the head model and at the same time the received signals are stronger than those received at other levels, for the same excitations (S2 L3 and S4 L3). Thus, the antenna radiates most efficiently along its centreline. Nonetheless, a drastic reduction of signal quality (which is quantified by low fidelity) and signal strength is noted when the probes are not along the centreline of the antenna. This scenario is vividly observed from the probe array measurements at S2 L3 and S4 L3. This is because the probes in higher/lower levels are in fact distant from the phase center of the antenna although the distances between the probes and antenna’s top surface are the same. Thus, the transmitted signals have to travel further from the phase center to higher/lower level probes and will thus encounter more losses and distortions. Similar observation can be concluded in case of the second set (L1, L3 and L5) (Fig. 4(e,g)). However, comparing the results of S2 L3 with S1 L3 and S4 L3 with S5 L3, it can be said that increasing the observation distance results in increasing losses and pulse distortions. Again, the propagated signals at the top levels of the head suffer from less losses and distortions. This observation is also noted from the total transient E_z_-field distributions inside the head model, illustrated in Fig. 4(h), where the top levels (S1 L1, S2 L2) are illuminated brighter than the lower levels (S4 L4, S5 L5). Again, it is observed from Fig. 4(h) that L3 is brightest in case of S3 scan. These numerical results confirm the suitability of this antenna arrangement for multi-slice imaging for accurate 3D localization of the hemorrhagic target. The implications of these observations are also confirmed in the imaging section.

### Frequency domain performance

**Figure 5:**
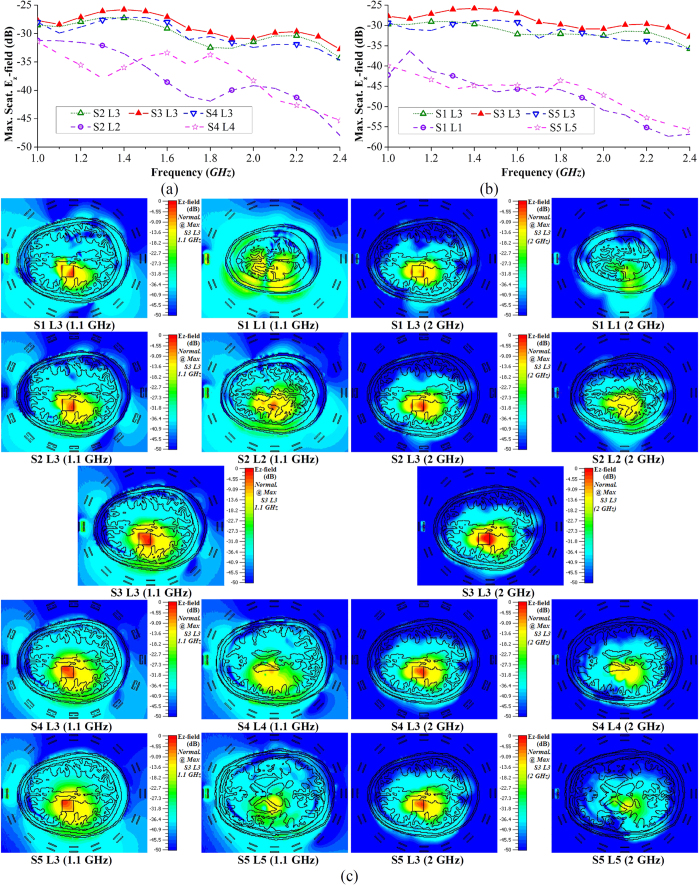
(**a**) The maximum scattered E_z_-field over the wide operating band at three different vertical levels with (**a**) 10 *mm* and (**b**) 20 *mm* separation where the ICH target is placed at the mid-level (L3) for the scanning. In the scanning array only antenna-1 (antenna in front of forehead) is excited. (**c**) The E_z_-field distributions of 2D cross-sections of different levels and for different excitations illustrating the scattered fields generated by the ICH target which is placed at L3 level.

The scattering from the previously discussed hemorrhagic target is calculated over the operating band. From the maximum scattering E_z_-field results presented in Fig. 5(a,b), it can be seen that the scattering fields vary over the band and they tend to go lower at higher frequencies. As the conductivity of the head tissues gradually increases over the frequency band, higher frequencies face higher losses and thus less amount of power penetrates towards the target. On the other hand, the scattering signals from the target are also reduced for the same reason. It is noted from Fig. 5(a,b) that as the array moves from one level to another, the fields received at a certain level is different in every scan. This indicates that the imaging scenario gradually changes in every scan depending on the height of the scanning level position. However, as the target is located at L3, the maximum scattering E_z_-field at L3 level for any scanning position is higher than those at the top (L1, L2) or bottom (L4, L5) levels, where the fields gradually decay with increasing distance from L3. Moreover, S3 L3, where L3 is located at the centerline of the S3 scanning, the maximum E_z_-field is seen to be higher than the other scanning locations (S2 L3, S1 L3, S4 L3, S5 L3). This is because the signals have to travel longer distance through the head to reach the hemorrhagic target, thus suffer from higher losses.

The maximum E_z_-field distributions inside the head model for 1.1 and 2 GHz are illustrated Fig. 5(c). It can be seen that at both frequencies, the maximum scattering from L3 occurs at S3 scanning, as L3 is located at the middle of S3. The scattering for other scanning positions gradually becomes lower for L3 (S2 L3, S4 L3 etc.). As a result, the field distributions of their scanning levels (S2 L2, S4 L4 etc.) also demonstrate gradual decrease in the scattered fields. Moreover, the scattering patterns are found different at different frequencies and also at different levels; some are collimated at one point and some are distributed over multiple points.

Extended analysis mentioned in Supplementary Figs S1, S2 and S3 demonstrate that depending on the excitation, the level of scattered E_z_-field also varies. It is noted that for the side excitations (antenna-5, −13), the distance of the target from the skin layer decreases and the scattered fields consequently increase, and thus makes the detection easier. However, the scattering patterns vary for different excitations and frequencies due to the heterogeneous head’s tissue distribution, which looks different from different scanning positions for different frequencies.

### Experimental detection results

To validate the detection efficacy of the multi-slice compact head imaging system, several experiments are performed on a realistic human head phantom. The data is collected accordingly from the system and an image of the head interior is generated using an improved confocal imaging algorithm.

### Imaging scenarios

A realistic human head phantom, which is based on MRI-derived 3D model, is used for the validation of the multi-slice head imaging system (Supplementary Fig. S4). Different parts and casts of the head phantom are 3D printed. Artificially built tissue mimicking materials, which accurately emulate the frequency dispersive properties of the tissues across the used band, are utilized to fill-up various brain tissues using the casts. The realistic human head fabrication process and recipes of the tissue emulating materials are detailed in refs 54 and 55. In this work, four different imaging scenarios to investigate the performance of the imaging system are presented. Firstly, a healthy head phantom is imaged to examine if the system is able to debar false positive detection. Subsequently, ICH targets of 2 × 2 × 0.5 cm^3^ in three different locations inside the head phantom are imaged to analyse the detection capability of the system. The targets are placed in shallow-right, deep-left, and deep-back positions. Targets are placed in different vertical positions (centred at 40, 30 and 20 *mm* away from the top head level) to investigate the effects of ICH scattering in multiple levels.

### Experimental Results

**Figure 6:**
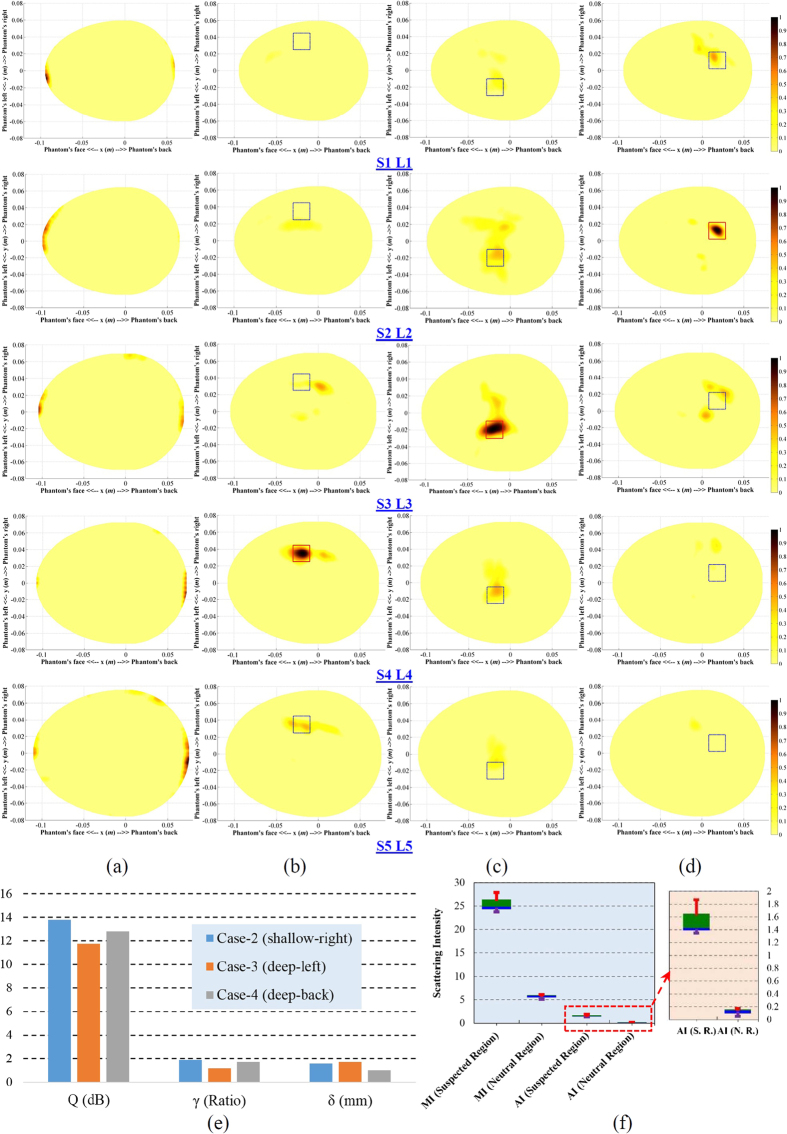
The reconstructed images of realistic human head phantom at five different levels for four different cases. (**a**) Healthy, (**b**) shallow-right, (**c**) deep-left, **(d)** deep-back positions. (**e**) The quantitative analysis results of the detected targets in the detected level. (**f**) The statistical analysis results of the maximum and average intensities in neutral and suspected regions. MI = maximum intensity, AI = average intensity, S. R. = suspected region and N. R. = neutral region. The green and blue boxes represent the 75^th^ and 25^th^ percentiles respectively and the transitional line between them presents the median. The red and violet lines accordingly state the maximum and minimum magnitudes.

Following data acquisition and processing, the images of the realistic human head phantom are reconstructed as depicted in Fig. 6. Five scans are presented for each of the four experimental setups. No significant scatterer is noted for the healthy head phantom (Fig. 6(a)), which makes it free from false positive detection. In this case, maximum scattering intensity is located near the skin layer. On the other hand, for the ICH affected head phantom (Fig. 6(b–d)), strong scatterers are located inside the suspected region. Maximum scattering intensities are found at level-4, -3 and -2 accordingly for the shallow-right, deep-left and deep-back target locations, which are the correct vertical scanning position (S4, S3 and S2 accordingly). Supplementary Fig. S5 demonstrates the sequential steps of the data acquisition, post-processing and image reconstruction of case-2 (deep-left target). It is seen that the threshold normalization process correlates the images for 3D localization of ICH target.

Supplementary Figs S6 and S7 illustrate the comparisons of using individual normalization of the reconstructed images with those of the threshold normalization. It is seen that for the healthy head phantom (Supplementary Fig. S6(a,b)), even individual normalization shows that the maximum scattering is located at the neutral layer. Since the threshold function (defined in Methods section), in this case, is *ρ* > 1, the maximum value of the neutral region is defined as the lowest thresholding value. The actual values of the maximum intensities for different scanned levels are mentioned in Supplementary Fig. S8(a). Very low discrepancies among the values are observed.

For the unhealthy head phantoms containing the ICH targets (Supplementary Figs S6(c,d) and S7), when the reconstructed images of each level are normalized to their individual maximum, they demonstrate one or multiple targets in different locations of the head. However, from Supplementary Fig. S8(b–d), it is evident that the layers have different maximum values in the suspected regions of different scanned levels and the maximum values of the actual target location are the highest in each experimental set. However, the maximum values of the neutral region show very low fluctuations.

Quantitative analysis is performed on the reconstructed images with maximum scatterers and the results are presented in Fig. 6(e). It is seen that the images yield high values of average signal to clutter ratio (*Q*), maximum signal to clutter ratio (*γ*) and accuracy indicator (*δ*). However, it is noted that the detection for shallow targets is easier, as the signal to clutter ratio indicators have higher values when compared to the deep targets. This is because the scattered signals in shallow position suffer comparatively from lower penetration losses. This phenomenon is also evident from the raw scattering intensity values of the experimental sets (Supplementary Fig. S8(b–d)). However, as low values of accuracy indicator are achieved, it can be said that all the experimental sets attain high accuracy of detection when using the proposed multi-slice head imaging system and its associated algorithm.

Statistical analysis is also performed on the reconstructed images of all the ICH affected head phantoms. It is noted that both of the maximum intensity (MI) and average intensity (AI) medians of the suspected regions of all the three experimental sets are around 5 times higher than those of the neutral regions. Again, it is observed that the MI and AI of the suspected region exhibit higher fluctuations compared to those of the neutral region. This is because the scattered energy, depending on the target position (shallow or deep), varies owing to the penetration losses, while the scattering noise of the neutral region has less fluctuations.

### Pilot volunteer study using the prototyped system

To apply the prototyped multi-slice head imaging system for pre-clinical volunteer study, the radiation safety of the prototyped system is checked through numerical simulations. Supplementary Fig. S9 illustrates the specific absorption ratio (SAR) simulation setup, which contains the previously discussed MRI-derived numerical head model and various results. In the mono-static system, only one antenna is active at a certain moment. Thus, SAR of the system is calculated by exciting one antenna at a time. In this case, four orthogonally positioned antennas (antenna-1, -5, -9, -13 (shown in Fig. 4(a))) are considered and excited with 0 dBm (1 mW) power, which is the typical power level transmitted from the antennas of the prototyped system. It is observed (from Supplementary Fig. S9(a)) that the calculated SAR values are significantly less than the IEEE defined radiation limit of 2 W/kg^56^,^57^ indicating the safe use of the presented system. The SAR distributions of the head model (from Supplementary Fig. S9(d–g)) illustrates that for different antenna excitations, maximum SAR values are located on the skin layer and concentrated at the closed point from the phase center location of the antennas.

**Figure 7:**
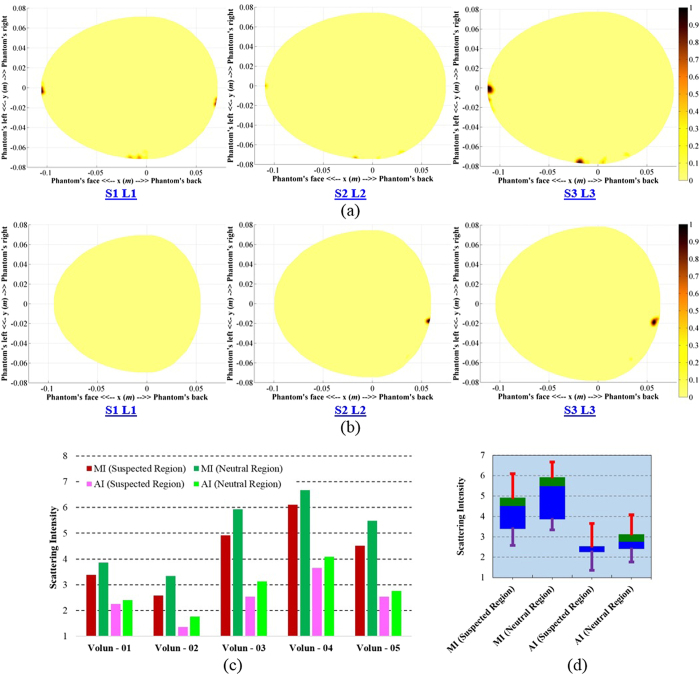
(**a**,**b**) The image formation results of two volunteers’ heads scanned at three different levels. (**c**) The maximum and average intensity comparisons between the suspected and neutral regions for five different volunteers. (**d**) The statistical analysis of the maximum and average intensities of neutral and suspected regions. The green and blue boxes represent the 75^th^ and 25^th^ percentiles, respectively, and the transitional line between them presents the median. The red and violet lines accordingly state the maximum and minimum magnitudes.

After verifying the safety issues of the prototyped imaging system, it is applied on healthy volunteers in preclinical trials. The heads of the volunteers are scanned at three levels having separations of 10 *mm*. The reconstructed images from two volunteers are depicted in Fig. 7(a,b). There is no target detected inside the suspected region and the maximum scattering is identified as imaging noise. It is clear that the reconstructed images of all levels are free from false positive detection. Supplementary Figs S10–S14 demonstrate the imaging comparisons between the reconstructed images with individual normalization and the threshold normalization. Threshold normalization reduces the noise level and makes it clear that most of the scattering signals come from the neutral region. The performed statistical analyses (Fig. 7(c,d)) of the reconstructed volunteer images also suggest that the skin reflection is slightly higher than those of the internal scattering from the brain portion due to the high contrast in the dielectric properties at the skin-air interface compared with the contrast between internal head tissues.

## Discussions

A compact and portable multilevel head imaging system prototype operating over the band 1–2.4 GHz is presented in this manuscript. The head imaging platform can be adjusted for different patients’ head shapes and sizes, and can be utilized to scan multiple levels of the head. The platform is fabricated using different 3D printed parts. Thus, with a choice of multiple low-cost 3D printing materials, a faster, accurate and unified manufacturing process can be guaranteed compared to the other head imaging prototypes^32^,^33^,^34^. 3D printing also allows the system designers to easily modify the system for any specific environment, e.g. bedside, ambulance etc. In this research work, the whole platform is fabricated using different 3D printed parts, except some minor components, like, nylon screws, which are commercially available in low cost. The imaging system consists of an array of compact sensing antennas which are connected to a switching network. A compact microwave transceiver is used for data acquisition. A laptop is used for data acquisition and signal processing purpose.

To satisfy the demands of microwave based head imaging system, an antenna, which is evolved from a typical folded dipole structure, is designed. The antenna functions over 83% fractional bandwidth at the center frequency of 1.7 GHz with directional radiation patterns over the whole band. Multiple techniques including slot-loading, folding and furling methods are applied to make the antenna compact. The overall volume of the antenna is 0.033 × 0.033 × 0.27 *λ*_*0*_^3^, which is 50% smaller than the previously reported directional, wideband antennas^19^,^20^ (Supplementary Table S1). This compact size of the antenna enables using 16 elements in the array surrounding the head with low mutual coupling between neighbouring antennas.

The minimum and maximum number of antennas for the head imaging can be calculated by the degrees of freedom (DOF) theory^58^,^59^,^60^. According to DOF, the imaging domain’s perimeter should be sampled at a minimum rate equal to one half of the wavelength in air (*λ*_*min*_) to be able to detect the target, Δ*φ* = *λ*_*min*_/2*a*, where *a* is the radius of the perimeter. In this case, the number of DOF of the head imaging system, and thus the maximum number of antennas required to collect the available information at the highest frequency and provide the best result is around 16. Moreover, it is noted that increasing the number of antennas over 16 brings the sensing antenna elements close to each other and increases the mutual coupling between the neighbouring elements without adding significant useful information.

Both of the near- and far-field radiation performances of the antenna in the time and frequency-domains confirm its potential for head imaging system. The whole system is validated by experimental imaging of various realistic scenarios of healthy and brain injured cases by means of a 3D realistic human head phantom. The acquired data is post-processed and the actual scattering signals are estimated by using a two staged calibration process. A modified DAS-based image processing technique relying on a point-of-entry dependent permittivity human head model is utilized to map 2D cross-sections of the head phantom. To accumulate the imaging results from multiple different levels, a thresholding is necessary to reduce the incurred imaging noise.

In ideal conditions, the two-stage calibration takes care of all reflection and background noise. However, in practice, there are several assumptions that might go wrong. Among them is the presumption that all switching network- antenna connections have the same characteristics. This assumption can be compromised as the qualities of all connectors, cables and antennas are not exactly the same. Although the first stage of calibration deals with this, in most cases some noise adds up in post-processing. Incorrectness of the assumption that each sensing antenna element is located at a constant distance from the boundary vector and that the skin layer has a uniform thickness can also be another source of noise in post-processing. The noise coherently gets added up at the skin layer. Nevertheless, the permittivity contrast of the internal brain parts also produces scattering signals. However, these scatterings are around 10–20% of the actual ICH target in case of brain injured patient.

The thresholding normalization process is very effective in improving the image quality of an unhealthy head as the skin reflection is considered the minimum threshold signal level. On the other hand, the maximum scattering intensity is selected as the maximum threshold level. For ICH affected unhealthy head phantoms, maximum scattering is found at the level where ICH target is located. This phenomenon confirms the numerical results of both time and frequency domain scattering characterizations (Figs 4 and 5). However, it is found from the images of the healthy head phantom that the residual skin scattering has the maximum value and thresholding normalization significantly improves the image quality. Compared to the previously reported DAS-based head imaging ICH detection results^32^,^33^,^34^, it can be concluded that the thresholding normalization can attain higher quantitative metrics. According to the comparison presented in Supplementary Table S2, it is noted that the proposed imaging system on average results images with at least 70% higher average signal to clutter ratio, 25% enhanced maximum signal to clutter ratio and with around 60% better ICH target localization.

Overall, from Fig. 6, it is observed that the shallow targets have higher scattering intensities than those of the deep targets. Nevertheless, among deep targets, top level located targets yield higher metric values than lower level targets. Due to the narrow shape of the human head at the top level, the signals have to penetrate less tissue, and are, consequently, confronted by less losses resulting in higher scattering intensities from the ICH target (Fig. 6(e)).

The numerical analysis of multi-level scattered Ez-field results (Fig. 5, Supplementary Figs S1, S2 and S3) and the reconstructed imaging results (Supplementary Figs S5, S6 and S7) indicate that in case of an ICH target, the scattered signal is radiated in a spherical manner even though the incident signal is coming from one direction excited by only one antenna (mono-static approach). Upon inception of the scattered signal at ICH target, the wave propagates through brain and demonstrates different scattered field patterns depending on the incident signal’s direction and the absorption of lossy head tissues. As a result, the scattered field is maximum at the centre level of ICH target (e.g. L3 in Supplementary Fig. S5), while each level has its own maximum. Therefore, if head scanning is performed at only one level other than the centre one and individually presented without comparing with the overall maximum value, the images still show one or multiple false targets (artefacts) with local maximum value. This can be observed at the stage before the application of threshold normalization in Supplementary Figs S5, S6 and S7. This is why, one of the important remarks that can be concluded here is that the previously reported 2D imaging systems are vulnerable to errors when compared to the presented multi-slice 3D imaging system.

It takes less than 2 seconds for the multi-slice system to collect data from one scan (Supplementary Table S2), while data acquisition of previous manual and mechanical rotational scanning system requires 1–4 minutes^32^,^33^,^34^,^35^. The time requirement for data acquisition of the multi-slice head imaging system is also lower when compared to other radar based breast cancer detection systems^61^,^62^,^63^. Microwave tomography based quantitative head imaging systems^36^,^37^,^38^ take comparable time to the proposed system for data acquisition. However, as mentioned in the introduction, the proposed radar based head imaging techniques demand less hardware resources and time (5–8 seconds depending on the size of head) for image reconstruction time owing to its faster signal and image post-processing capability compared to microwave tomography (from tens of minutes to hours^40^ depending on the required imaging accuracy). Unfortunately, time is critical for ICH affected patients^1^,^2^,^3^,^4^,^5^,^6^. Nevertheless, the imaging resolution of radar based imaging system^34^ (less than 10 *mm*) is comparable to the tomography based head imaging system^35^,^36^,^38^,^39^ (5–7 *mm*). This resolution is higher when compared to other portable imaging modalities^9^,^10^,^11^,^12^,^13^. Thus, the prototyped system can effectively be used as on-site and rapid diagnostic solution for multi-slice head imaging applications. It is worth to mention that the resolution is directly related to the operating frequencies of the system, which is theoretically limited by the size of sensing antennas. Hence, designing compact antennas can open new doors for advanced microwave imaging^30^,^31^,^32^,^33^,^34^,^35^.

The radiation safety of the imaging system is verified and the system is employed in pilot volunteer study. Five different healthy volunteer head images are presented for three different levels. It is observed that the conclusions from the healthy human head phantom are also true for the volunteer data. All the images are found free from false positive responses. This is an important criterion for any medical diagnostic solution.

**Figure 8:**
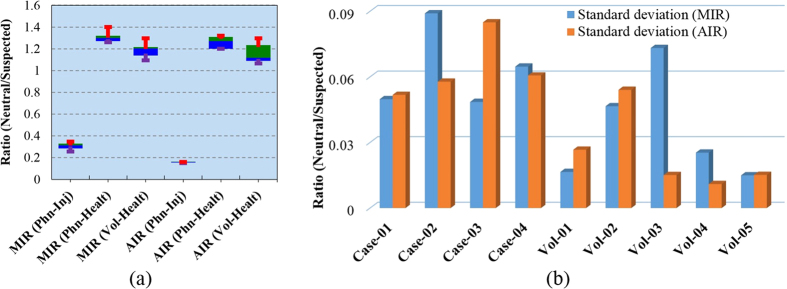
(**a**) The comparative view of the statistical analyses among the MIR and AIR values of the healthy head phantom and volunteer head, and unhealthy human head phantoms. MIR = maximum intensity ratio and AI = average intensity ratio. The green and blue boxes represent the 75^th^ and 25^th^ percentiles, respectively, and the transitional line between them presents the median. The red and violet lines accordingly state the maximum and minimum magnitudes. (**b**) The standard deviation results of each individual phantom and volunteer cases in terms of the intensity ratios of neutral and suspected regions.

Extended comparisons between different statistical analyses of the four phantom measurement cases and five volunteer study data are demonstrated in Fig. 8. It can be seen that the maximum and average intensity ratios (MIR and AIR) of the healthy scenarios are more than 1. Nevertheless, the rations are very close to each other, which confirm the imaging similarities between healthy realistic head phantom and volunteer heads as well as among different head sizes and shapes. However, medians of MIR and AIR values in case of brain injured phantom are around 0.3 and 0.16, respectively, indicating huge differences between healthy and unhealthy heads.

The maximum and minimum values of the MIR and AIR are limited up to 15% of the median values. This overall low level of fluctuation indicates the firmness of the image processing algorithm. The standard deviation of MIR and AIR ratios for the individual cases are demonstrated in Fig. 8(b). The very low standard deviation values indicate that the MIR and AIR in case of healthy and unhealthy patients are consistent when compared with different levels of each case.

## Methods

### Data acquisition system

To measure the scattering performance of the imaged head, data acquisition of the proposed imaging system comprises both software and hardware parts. The data acquisition is performed in five main stages: (1) System initialization and customization, (2) system calibration, (3) frequency scanning, (4) array scanning and (5) data storage. Automated data acquisition software is developed using MATLAB environment for data collection. Firstly, the controlling PC, microwave transceiver and switching network are switched on and all cable connections are checked accordingly. The positions of the antenna elements in the array are adjusted using the flexible imaging platform. The data acquisition software is initialized and the various parameters, e.g. transmitting power (*P*_*r*_), operating frequencies (*f*_*l*_, *f*_*h*_), scanning points (*M*) are imported inside the software. In the calibration stage, the microwave transceiver is calibrated up to the point of antenna feeding MCX connection through the switching network. The standard calibration system including open-short-load connections is adopted. This calibration gets rid of the effects of the switching system and long connecting cables which otherwise reduce the efficiency of the system. Afterwards, the frequency scanning is performed over the operating band using the data acquisition software to enable frequency scanning measurements of all 16 antenna elements in the array. The collected data is stored for processing.

### Numerical analysis of electromagnetic characteristics of the head

To analyse the electromagnetic characteristics of the human head when using the proposed multi-scan head imaging system, the imaging system is numerically analysed in finite difference time domain (FDTD) based electromagnetic software CST Microwave Studio (CST MWS). An MRI-scan based numerical human head model^64^ is imported accordingly and different tissues are assigned with individual frequency dependent electrical properties^65^ (permittivity and conductivity) for the operating band using a four-pole Debye model^66^:


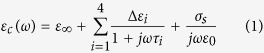


here, *ε*_*c*_ is the complex relative permittivity as a function of the angular frequency (*ω*). The permittivity of free-space (*ε*_0_), relative permittivity at infinity (*ε*_∞_), i^th^ relaxation time (*τ*_*i*_), magnitude of i^th^ dispersion (Δ*ε*_i_) and static conductivity (*σ*_*s*_) vary depending on the tissue type within the head. A brain injury imitating the hemorrhagic scenario of 2 × 2 × 0.5 cm^3^ size is placed inside the numerical model, and attributed with dispersive electrical properties of blood.

Time-domain signals are collected in multiple levels using multiple array of E-field probes and the analysis are performed following the metrics of refs 32 and 51. The frequency domain analysis of the scattering characteristics from a hemorrhagic target is necessary for understanding the working principle of the proposed mono-static multi-slice head imaging system. The electromagnetic scattering of a target from a non-magnetic, dielectrically heterogeneous domain at a measurement location of ***r***, can be reliably modelled^67^ by the following volume integral equation:


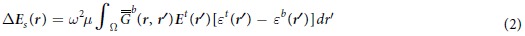


where Δ***E***_*s*_ is the total scattered field representing the total observed field difference between the target environment and the background environment, and Ω is the investigated region. The Green’s dyadic function 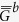 provides the field inside the head without the target. The relative permittivity of the target and background are denoted by *ε*^*t*^ and *ε*^*b*^ accordingly. ***E***^*t*^ is the total incident electric field due to the source antenna in the unhealthy head environment. 3D finite-difference time-domain (FDTD) based software CST MWS (Microwave Studio) in a realistic environment is utilized for the calculations.

### Signal and image processing algorithm

Data acquisition from the antennas is performed at multiple vertical levels (*L*) of the head phantom. In this study, *L* = 5 different levels at *D* = 10 mm separations are scanned, covering the brain portion of the head. Since the radii of the head vary from one patient to another, the positions of the antennas depend on the structure and shape of the patient’s head. Typically, the human brain is double parabolic shaped and symmetrical at mid-sagittal plane of the head^68^,^69^. Each level’s set of data contains reflection coefficient measurements (***Γ***_*n*_(*f*_*m*_), *n* = 1 to *N*, *m* = 1 to *M*) from *N* = 16 antennas over the band of 1–2.4 GHz. In order to apply the image processing algorithm, these frequency domain signals are converted to time domain (***T***_*n*_(*t*_*i*_)) using the inverse discrete Fourier transformation.


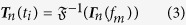



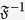
 represents the M × M matrix where the (n, k)th entry is 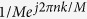, and *k* is the wave number.

In order to extract the scattered signals from the brain injury target, a two-stage calibration is utilized. The first calibration is used to trim the effects of the radiating antenna and the surround ones. In the previous single antenna based system^32^,^33^,^34^, this stage is not required, because of the presence of only one antenna. Since an array of antennas is used in the system, even though only one antenna is active and others are match terminated at a certain moment, the mere presence of other antennas, especially the neighbouring antennas, scatters some signals. To get rid of these scattering signals, one set of measurement (***Γ***_*empty*_(*f*_*m*_)) is performed with the same setup without the presence of the imaged head and consequently converted to time domain (***T***_*empty*_(*t*_*i*_)). This set is subtracted from the measured data and the actual scattering from the head without the effects of scattered signals from other antennas is obtained.


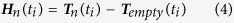


The second calibration is performed to acquire the target’s scattered signals (***S***_*n*_(*t*_*i*_)) from the actual scattering signals of the head (***H***_*n*_(*t*_*i*_)). In this case, the adjacent average subtraction technique, which considers the woody average of the adjacent antenna signals (***H***_*n*_(*t*_*i*_)), is deducted from the head scattering signals.


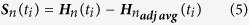


A confocal imaging algorithm based on a modified delay-and-sum (DAS) technique is employed for the image reconstruction purpose. An imaging area of 0.3 × 0.3 *m*^2^ is defined with a 0.5 × 0.5 *mm*^2^ pixel resolution. The outline of the human head is calculated from the scale of the antenna mount array and is defined by the boundary vector, ***B***_*j*_, *j* = 1 to *J*, is the border circumference at the skin-air interface in Euclidean space defined by *x* and *y* coordinates. In order to estimate the optimal propagation path from the antenna to an arbitrary point (***p***) of interest inside the head, Fermat’s principle is adopted by calculating minimum propagation route from all the possible routes (***d***_*j*_) from the antenna (***A***_*n*_) at free space to the boundary vector (***B***_*j*_) following the propagation paths from ***B***_*j*_ to the investigated point.


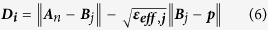


Because of the highly heterogeneous distribution of the human head tissues, a point of entry based permittivity model is used which is proven to have imaging advantages^33^ compared to the traditional constant permittivity models^32^.


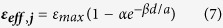


Using a similar procedure presented in ref. 33, the values for a typical human head model and the utilized antenna over the operating band of 1–2.4 GHz in a realistic simulation environment is estimated as, *ε*_*max*_ = 41, *α* = 0.7 and *β* = 6.3. Owing to the utilized mono-static approach, the wave has to travel to and from each points; therefore, the propagation time of the optimal route is multiplied twice to determine the actual delay (***τ***_*actual*_) of the target’s response. Finally, an image of the backscattered signals’ intensity is reconstructed by calculating the coherent summation of all responses.


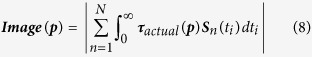


In case of true scattering point, the scattering intensity will be high, as in case of hemorrhage. On the other hand, small values will result from the wrong assumption of the hypothetical point indicating an imaging noise. 2D maps of the scattered energy from the head in multiple levels are reconstructed.

In delay-and-sum based back-projection approach, the absolute values of the image after image reconstruction do not provide other useful information regarding the images^32^,^33^,^48^. Hence, typically, cross-sectional 2D images are normalized to individual highest intensity. However, a scattering threshold is required for the proposed multi-slice head imaging system to differentiate between the healthy and unhealthy cases.

Due to the fact that the ICH does not occur in the skin or skull region^1^,^2^,^3^,^4^,^5^,^6^, the skin and skull tissues (which are about 10 *mm* thick) are considered as a neutral region (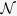), whereas the rest head interior is considered as the suspected region (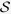). According to DAS algorithm, ICH targets are expected to scatter strongest signals in unhealthy cases. Therefore, in those cases, the system is expected to detect the strongest scattrer in the suspected region. On the other hand, for healthy cases, the suspected region holds the maximum of the scattered signals. Again, it is concluded from Fig. 5 that the maximum scattered signal from an ICH target, is located when scanning the level where it is located. Thus, in both cases, the images can be normalized with the maximum of the all 2D scans, ***Z***_*L*_, where *L* = 1 to 5 the number of scanned levels.


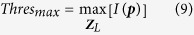


However, in order to determine the threshold, a function, *ρ* is defined as the ratio of maximum of the neutral region to that of the suspected region for scattering signals from all levels.


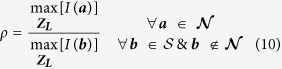


In case of, *ρ* > 1, 
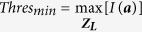
, while for *ρ* < 1, 
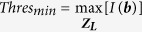
.

Finally, the multi-slice images are normalized with respect to the threshold values. The resulted images illustrate 3D localization of the potential scatterer in multi-slice scenario.

### Quantitative analysis of reconstructed images

The average signal to clutter ratio function, *Q* is defined by the following equation,


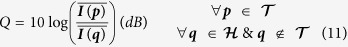


where the cross-sectional region of the head and target regions are, respectively, defined as 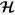 and 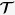. The easy identification of ICH targets is suggested by high *Q* values.

The maximum signal to clutter ratio (*γ*) is defined as,


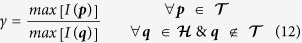


Strong clutter inside the image compared to the actual ICH target can be identified as the value of *γ* falls closer to unity.

The accuracy indicator (*δ*) is defined as,


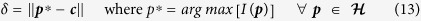


Perfect identification of the target is demonstrated when *δ* is zero.

### Pilot volunteer study

The prototyped multi-slice head imaging system for pre-clinical volunteer study, which is a common practice to prepare a diagnostic system for clinical environment^25^,^41^. The underlying aim of the pilot volunteer study is to verify the conclusions of the realistic phantom measurements as well as to investigate the possibility of building a database of healthy and unhealthy patients which can be utilized as a reference. The performed preclinical trials are executed following the guidelines and protocols of the Medical Research Ethics Committee (MREC) of the University of Queensland, which approved the preclinical microwave head imaging system and its measurement procedure (Clearance Number: EC201407MOB). Informed consents of all volunteers are obtained before the study.

## Additional Information

**How to cite this article**: Mobashsher, A. T. and Abbosh, A. M. On-site Rapid Diagnosis of Intracranial Hematoma using Portable Multi-slice Microwave Imaging System. *Sci. Rep*. **6**, 37620; doi: 10.1038/srep37620 (2016).

**Publisher’s note:** Springer Nature remains neutral with regard to jurisdictional claims in published maps and institutional affiliations.

## Supplementary Material

Supplementary Information
