# Supplementary material for: On-site Rapid Diagnosis of Intracranial Hematoma using Portable Multi-slice Microwave Imaging System
Source: Sci Rep. 2016 Nov 29;6:37620. doi: 10.1038/srep37620 (PMC5126641; doi:10.1038/srep37620)
Supplement: Supplementary Information [file srep37620-s1.pdf]

Supplementary Information

On-site Rapid Diagnosis of Intracranial Hematoma using Portable Multi-slice Microwave Imaging System

Ahmed Toaha Mobashsher*, and A.M. Abbosh
School of ITEE, The University of Queensland, St Lucia, 4072, Brisbane, Australia
*Email: a.mobashsher@uq.edu.au

Supplementary Table S1: Comparison of the proposed novel antenna with other wideband antennas

Antennas	Fractional bandwidth with respect to center frequency (%)	Relative antenna dimensions (Compared to lower frequency wavelength)			Additional ground plane?
		Length	Width	Height	
[42]	77	0.33	0.25	0.07	No
[67]	83	0.66	0.17	0.08	Yes
[68]	128	0.57	0.29	0.19	No
[69]	109	0.26	0.11	0.05	No
[70]	100	0.27	0.2	0.07	No
[71]	57	0.24	0.1	0.05	No
[72]	27	0.02	0.05	0.12	No
[73]	63	0.29	0.12	0.06	No
[74]	67	0.29	0.08	0.04	No
This Work	83	0.27	0.033	0.033	No

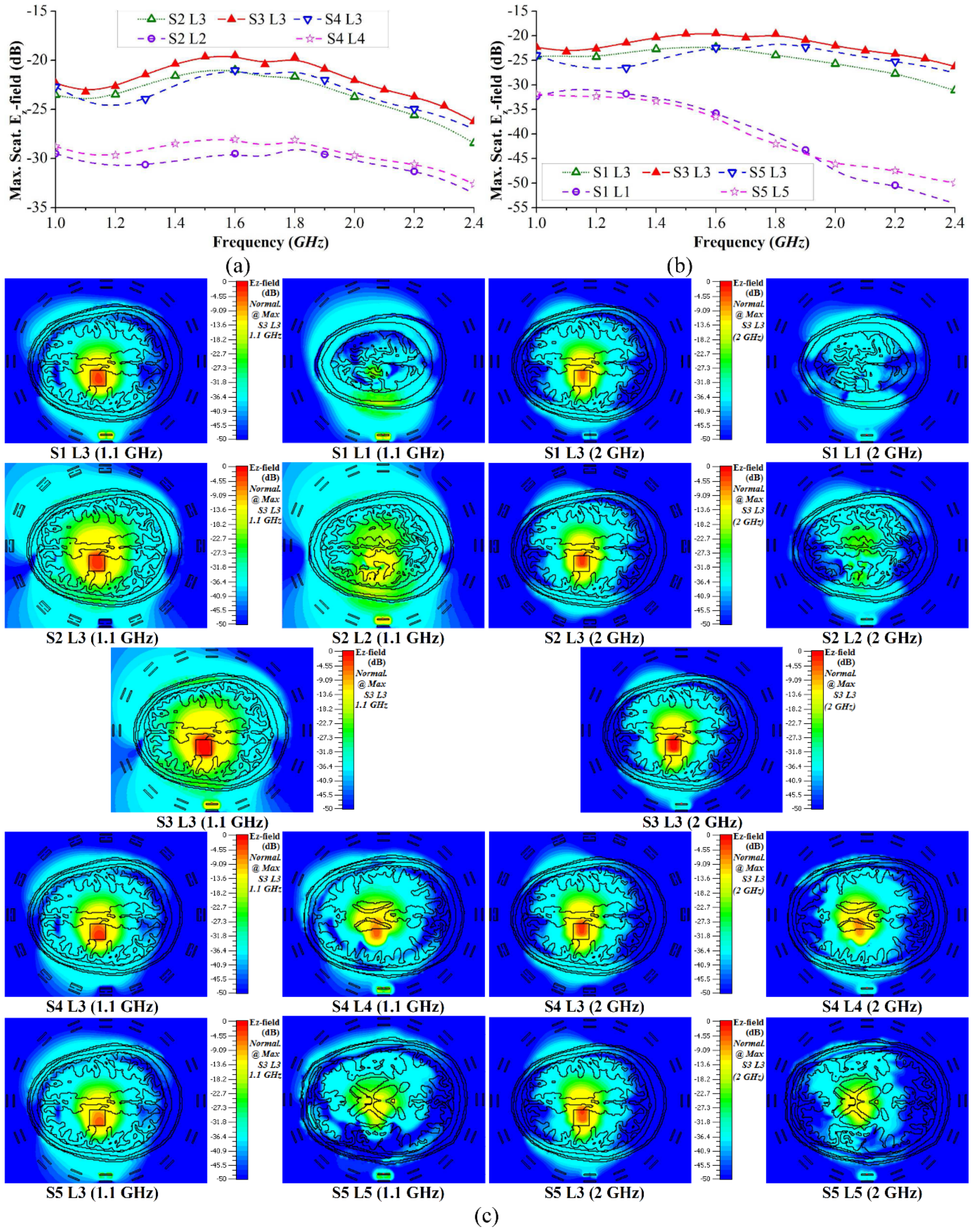

Supplementary Fig. S1. (a) The maximum scattered E_z-field over the wide operating band at three different vertical levels with 10 *mm* separation where the ICH target is placed at the mid-level (L3) for the scanning. In the scanning array only antenna-5 is excited. (b) The scattered E_z-field for antenna-5 excitation at three different vertical levels with 20 *mm* separation where the ICH target is placed at the mid-level (L3) for the scanning. (c) The E_z-field distributions of 2D cross-sections of different levels and for different excitations illustrating the scattered fields generated by the ICH target which is placed at L3 level.

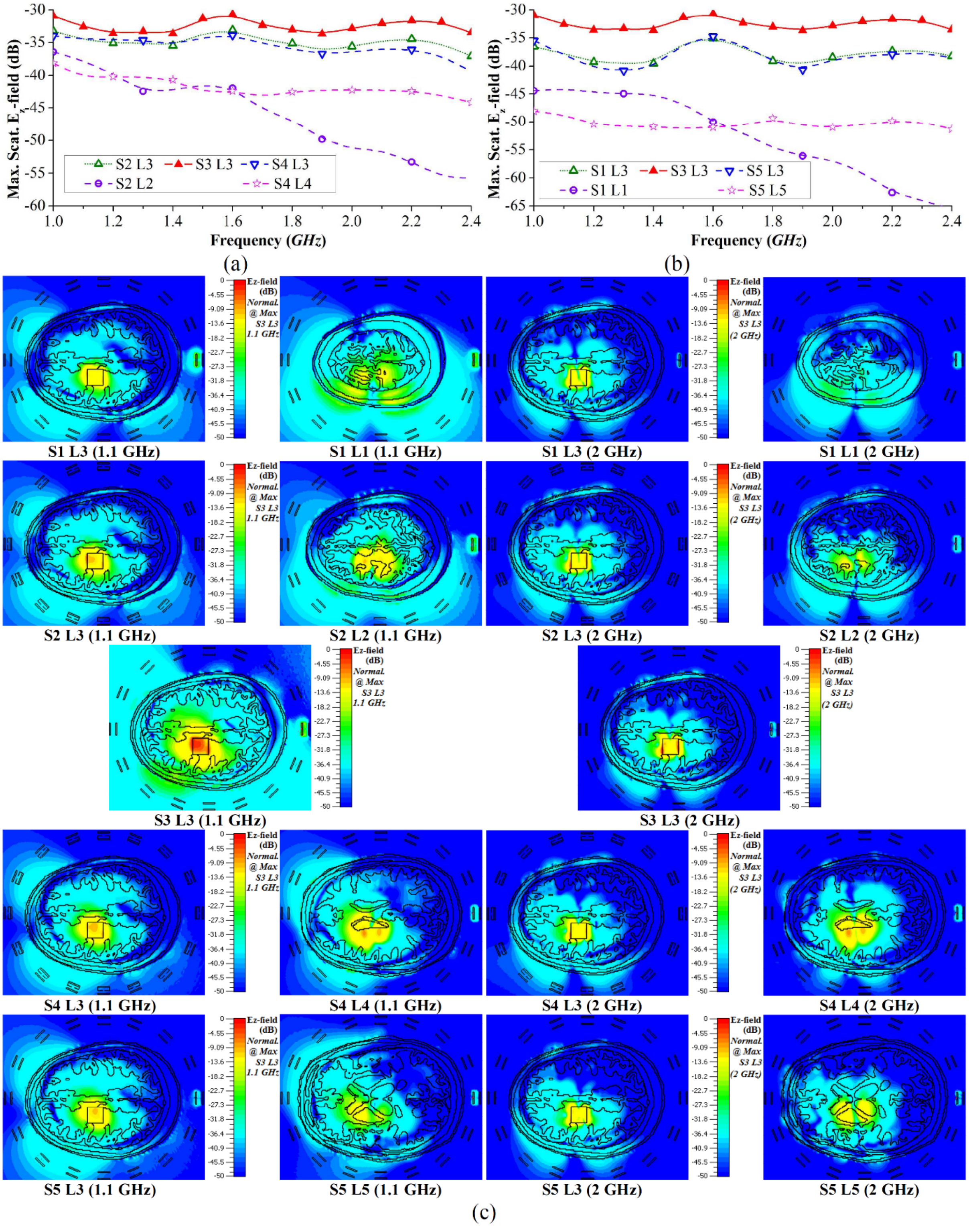

Supplementary Fig. S2. (a) The maximum scattered E_z-field over the wide operating band at three different vertical levels with 10 *mm* separation where the ICH target is placed at the mid-level (L3) for the scanning. In the scanning array only antenna-9 is excited. (b) The scattered E_z-field for antenna-9 excitation at three different vertical levels with 20 *mm* separation where the ICH target is placed at the mid-level (L3) for the scanning. (c) The E_z-field distributions of 2D cross-sections of different levels and for different excitations illustrating the scattered fields generated by the ICH target which is placed at L3 level.

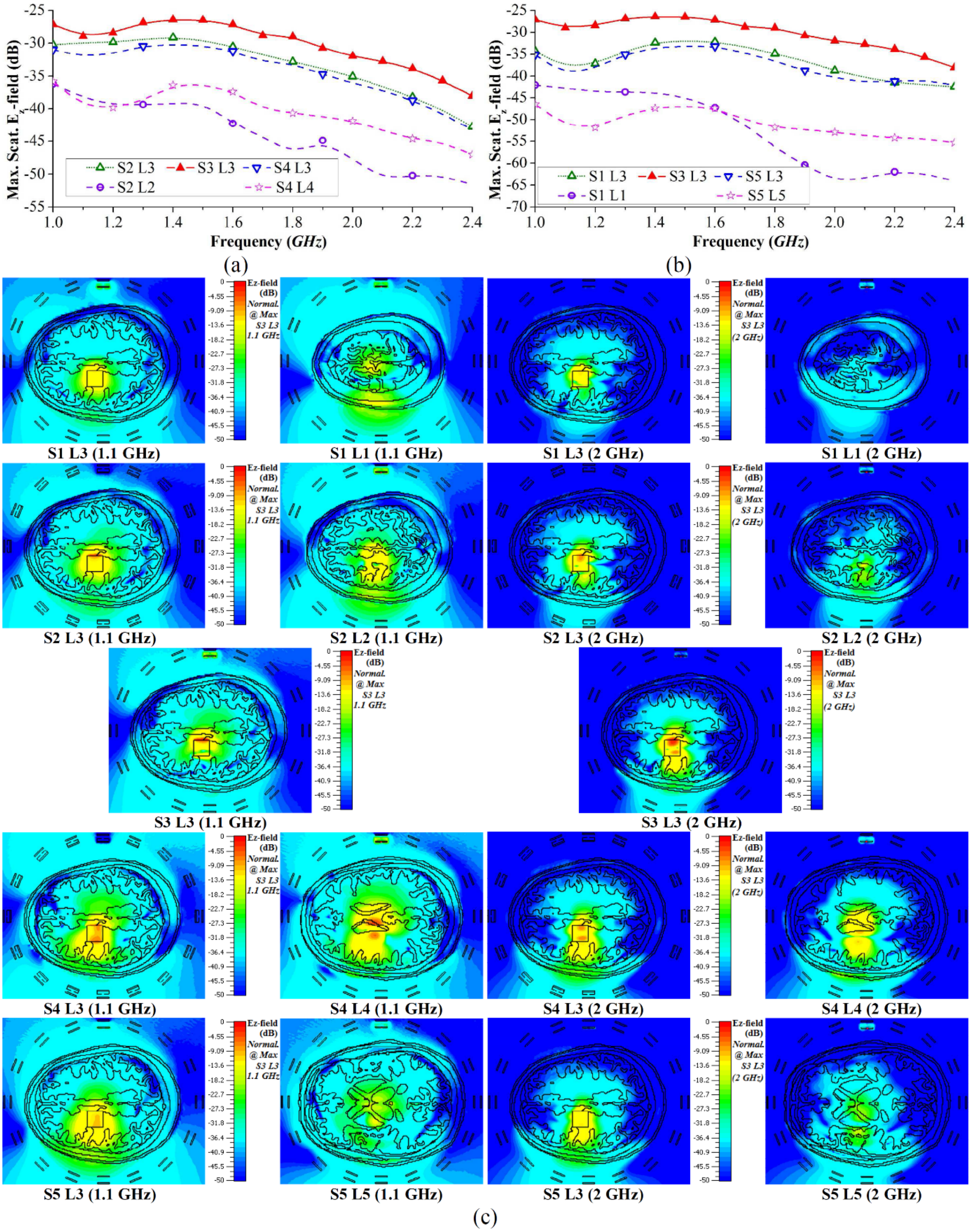

Supplementary Fig. S3. (a) The maximum scattered E_z-field over the wide operating band at three different vertical levels with 10 *mm* separation where the ICH target is placed at the mid-level (L3) for the scanning. In the scanning array only antenna-13 is excited. (b) The scattered E_z-field for antenna-13 excitation at three different vertical levels with 20 *mm* separation where the ICH target is placed at the mid-level (L3) for the scanning. (c) The E_z-field distributions of 2D cross-sections of different levels and for different excitations illustrating the scattered fields generated by the ICH target which is placed at L3 level.

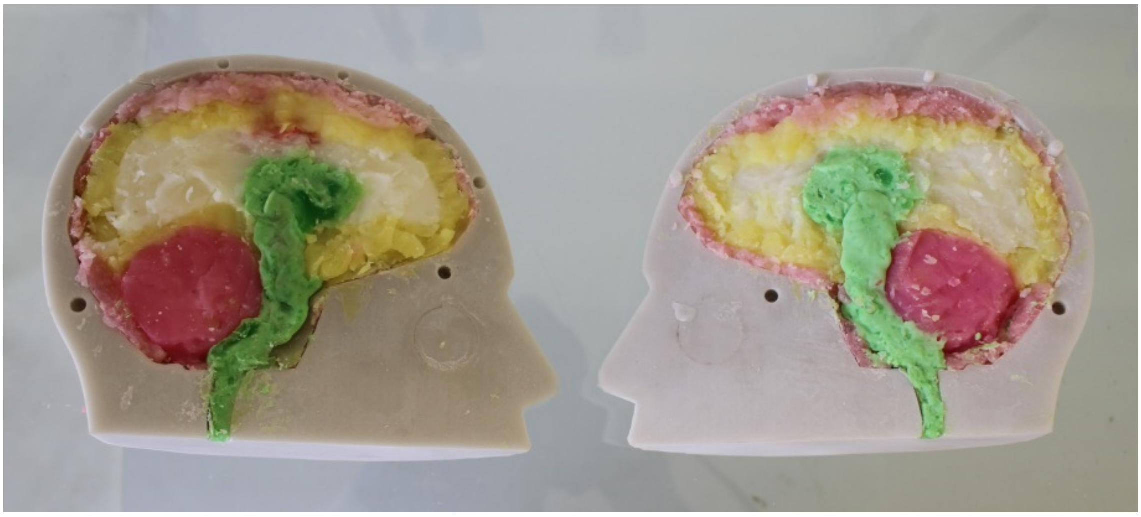

(a)

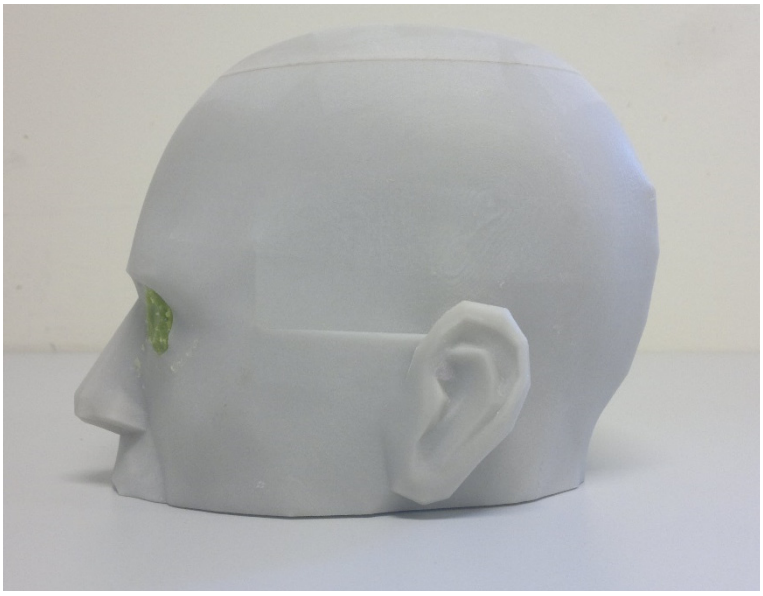

(b)

Supplementary Fig. S4. Photographs of the utilized artificial human head phantom. (a) Two completed halves of the head phantom before interlocking them to each other. The exterior of the head phantom is 3D printed using a high permittivity ABS plastic material using selective laser sintering (SLS) technique. The intracranial tissues of the head are fabricated using different tissue mimicking artificial materials emulating respective dielectric properties of actual individual brain tissues over a wide bandwidth of 0.5-4 GHz. The details of the fabrication process are described in ref 54, 55. (b) Side view of vertically laid the fully fabricated head phantom.

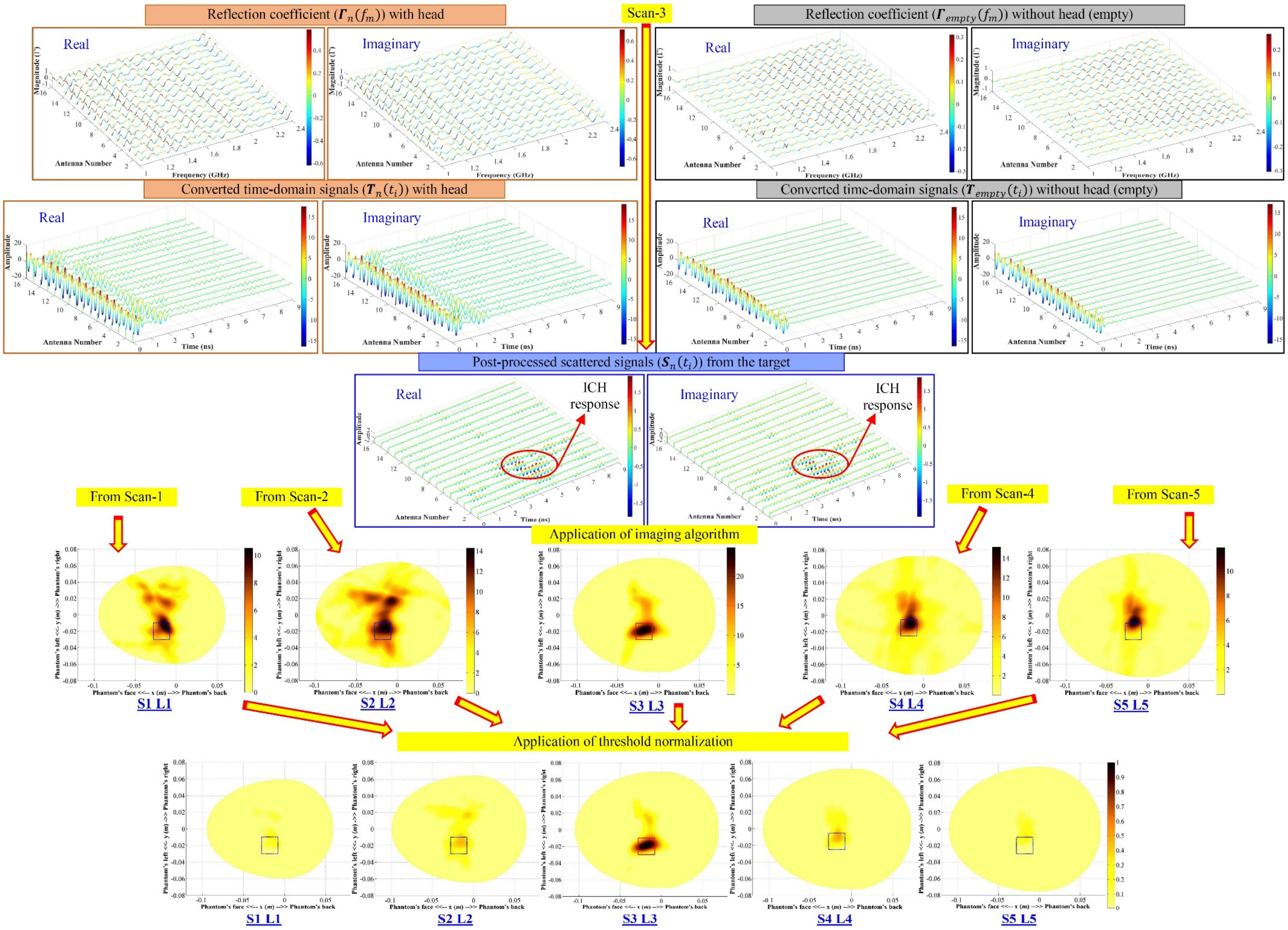

Supplementary Fig. S5.Step-by-step illustration of data processing and image reconstruction from multiscan head imaging system using back-projection algorithm and threshold normalization.

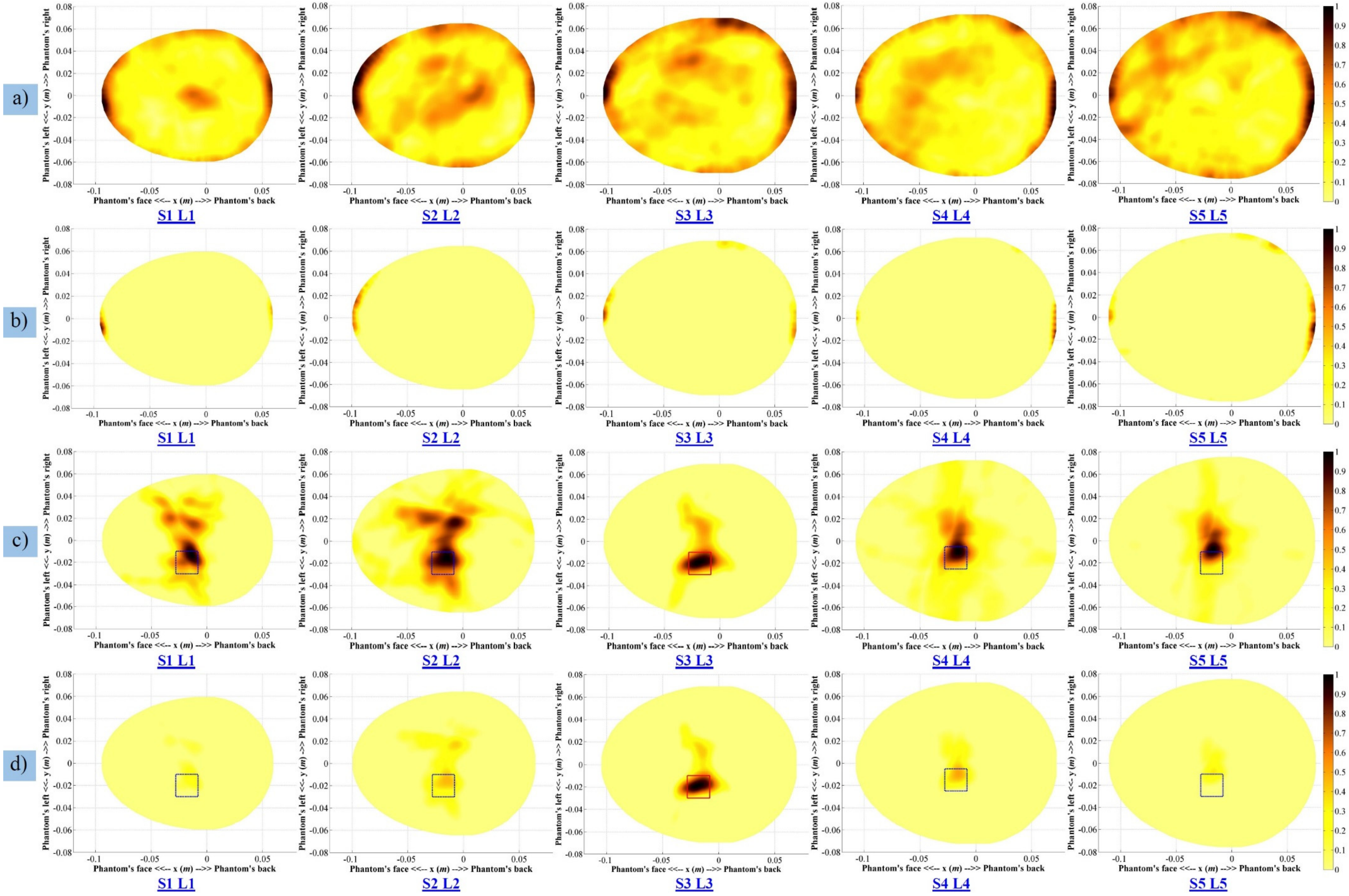

Supplementary Fig. S6. The reconstructed images of five different levels of healthy realistic human head phantom after (a) normalization with respect to individual maximum and minimum of each level, and (b) threshold normalization. The reconstructed images of five different levels of unhealthy realistic human head phantom with target at deep-left location after (a) normalization with respect to individual maximum and minimum of each level, and (b) threshold normalization.

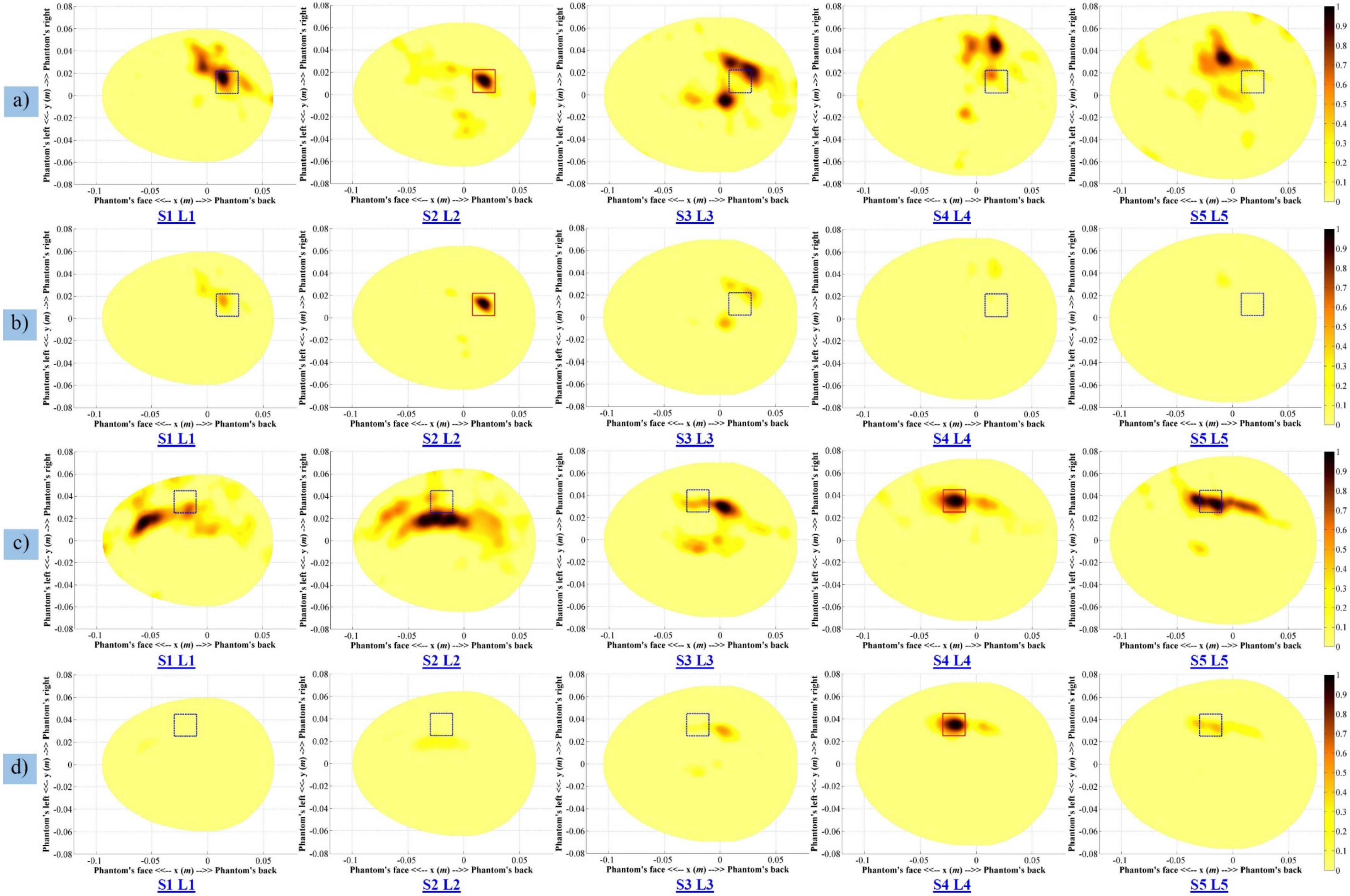

Supplementary Fig. S7. The reconstructed images of five different levels of unhealthy realistic human head phantom with target at deep-back location after (a) normalization with respect to individual maximum and minimum of each level, and (b) threshold normalization. The reconstructed images of five different levels of unhealthy realistic human head phantom with target at shallow-right location after (a) normalization with respect to individual maximum and minimum of each level, and (b) threshold normalization.

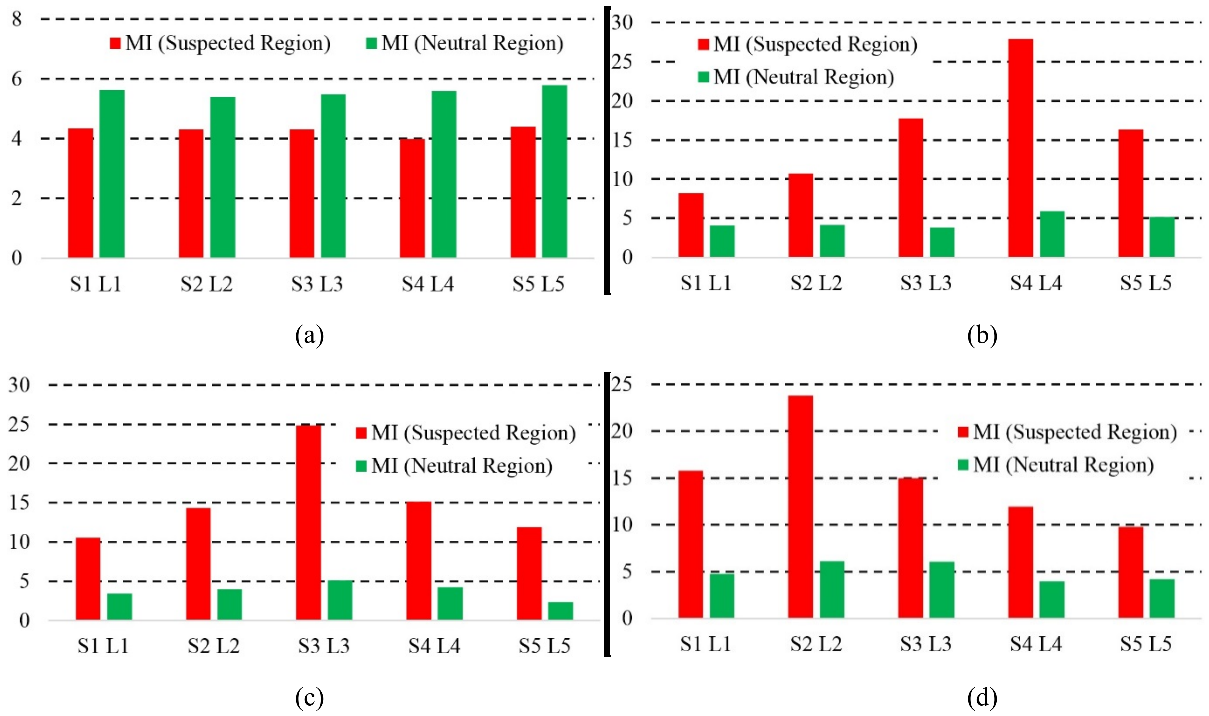

Supplementary Fig. S8. The raw maximum intensity values of different scanning levels for four different cases: (a) healthy, and unhealthy with ICH targets at (b) shallow-right, (c) deep-left, (d) deep-back positions.

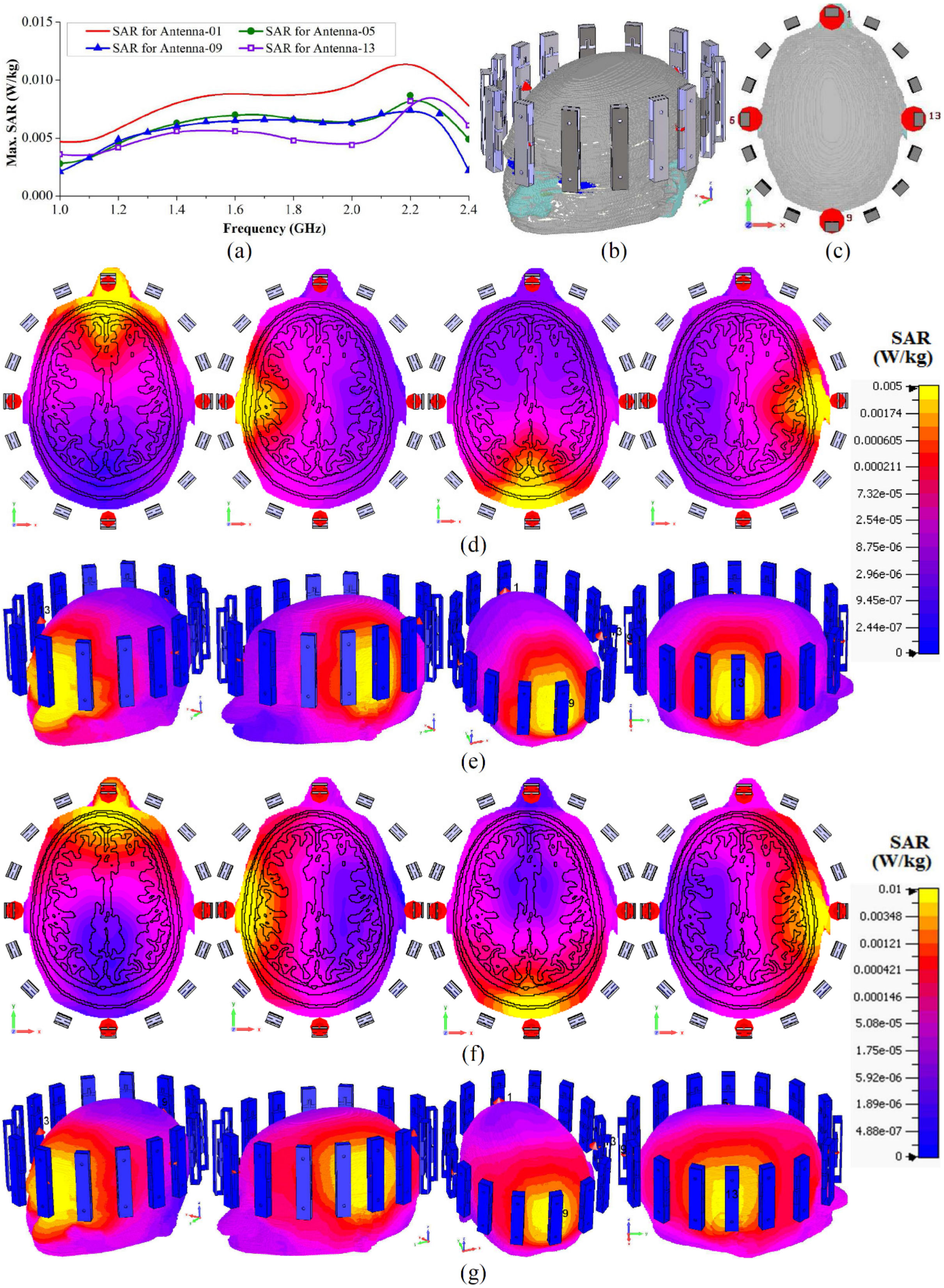

Supplementary Fig. S9. (a-c) The maximum SAR values of realistic human head model for antenna-1, -5, -9 and -13, with the illustration of simulation environment and antenna positions. The SAR distributions of the head phantom at 1.1 GHz for antenna-1, -5, -9 and -13, respectively illustrating (d) cross-sectional view and (e) the 3D view. The SAR distributions of the head phantom at 2 GHz for antenna-1, -5, -9 and -13, respectively illustrating (d) cross-sectional view and (e) the 3D view.

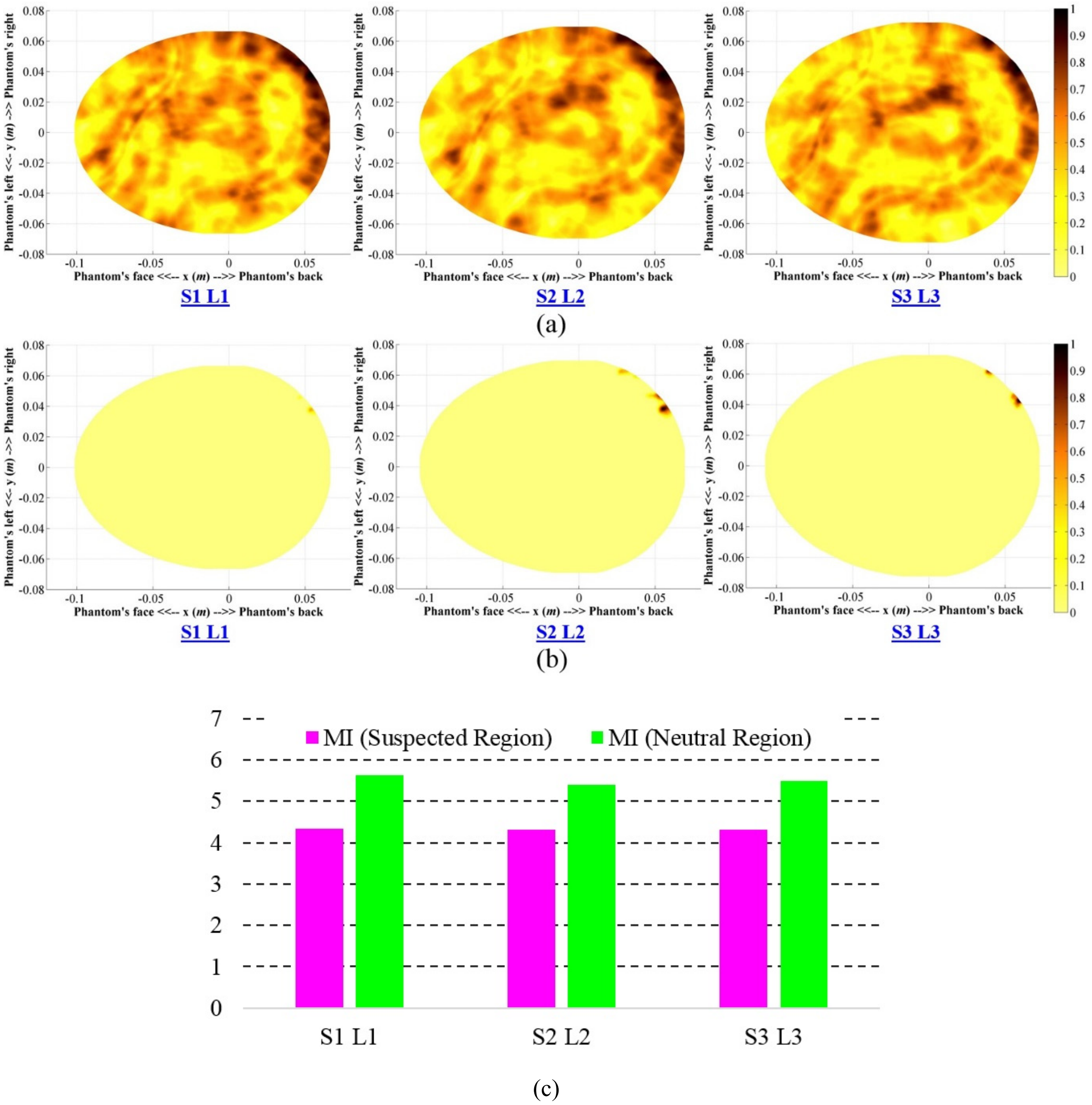

Supplementary Fig. S10. The reconstructed images of three different levels of healthy human volunteer-01 after (a) normalization with respect to individual maximum and minimum of each level, and (b) threshold normalization. (c) The raw maximum intensity values of the suspected and neutral regions at different scanning levels.

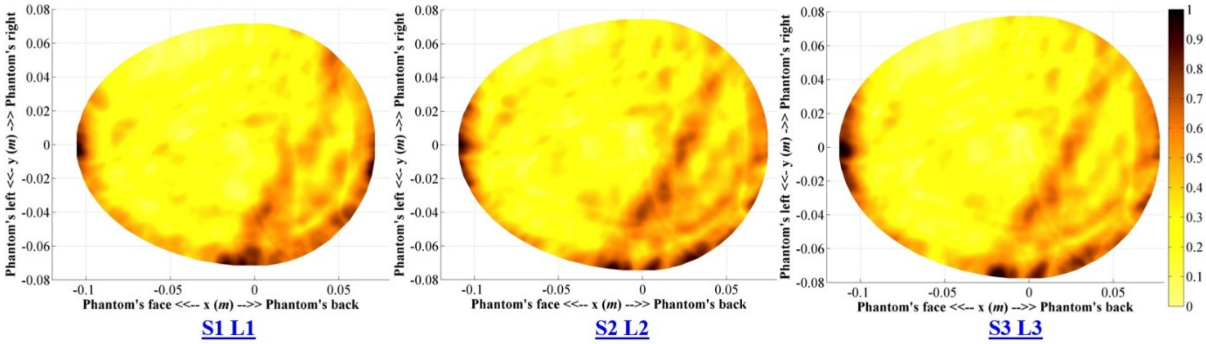

(a)

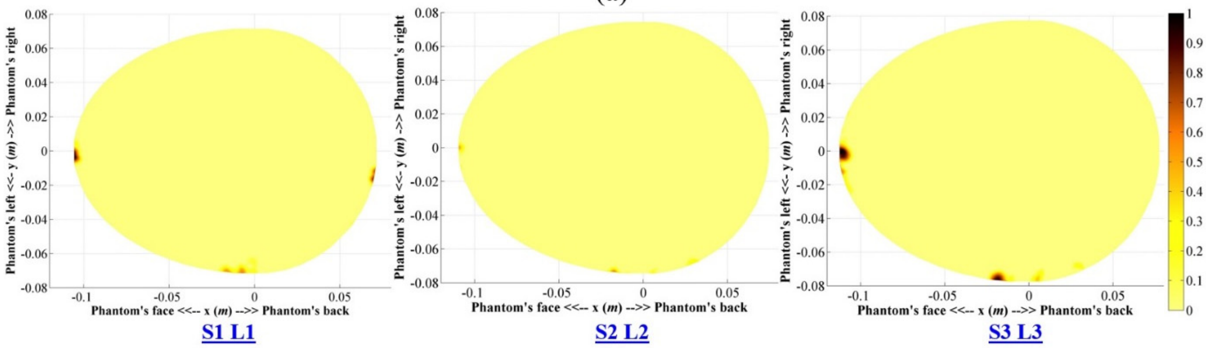

(b)

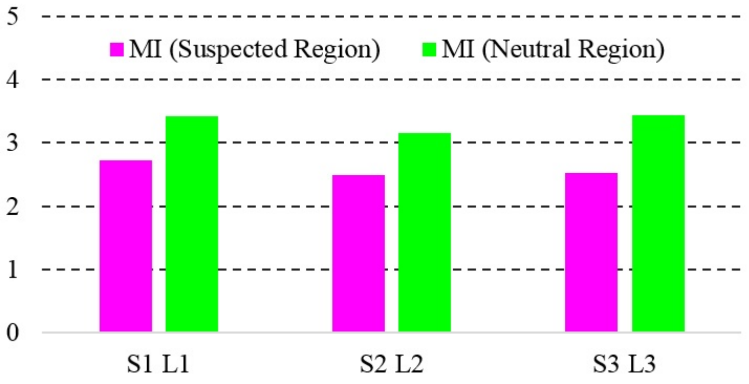

(c)

Supplementary Fig. S11. The reconstructed images of three different levels of healthy human volunteer-02 after (a) normalization with respect to individual maximum and minimum of each level, and (b) threshold normalization. (c) The raw maximum intensity values of the suspected and neutral regions at different scanning levels.

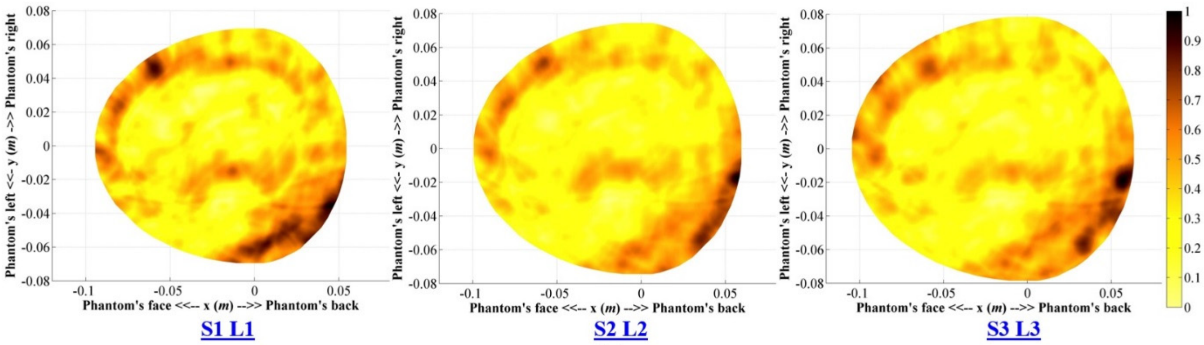

(a)

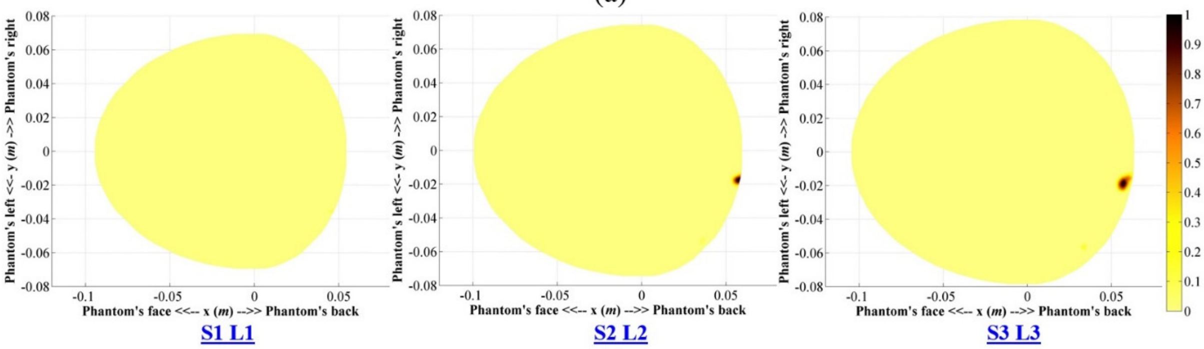

(b)

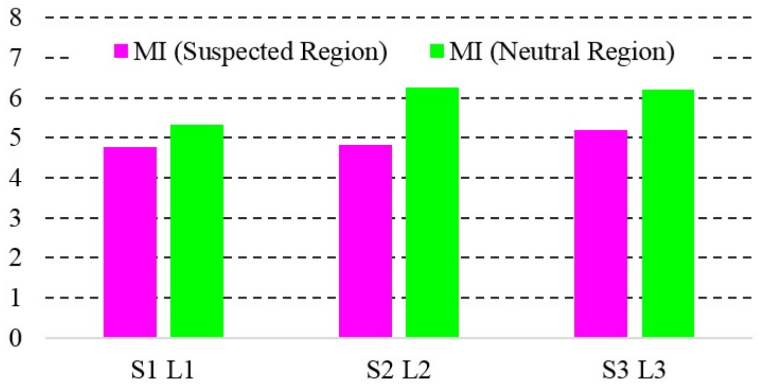

(c)

Supplementary Fig. S12. The reconstructed images of three different levels of healthy human volunteer-03 after (a) normalization with respect to individual maximum and minimum of each level, and (b) threshold normalization. (c) The raw maximum intensity values of the suspected and neutral regions at different scanning levels.

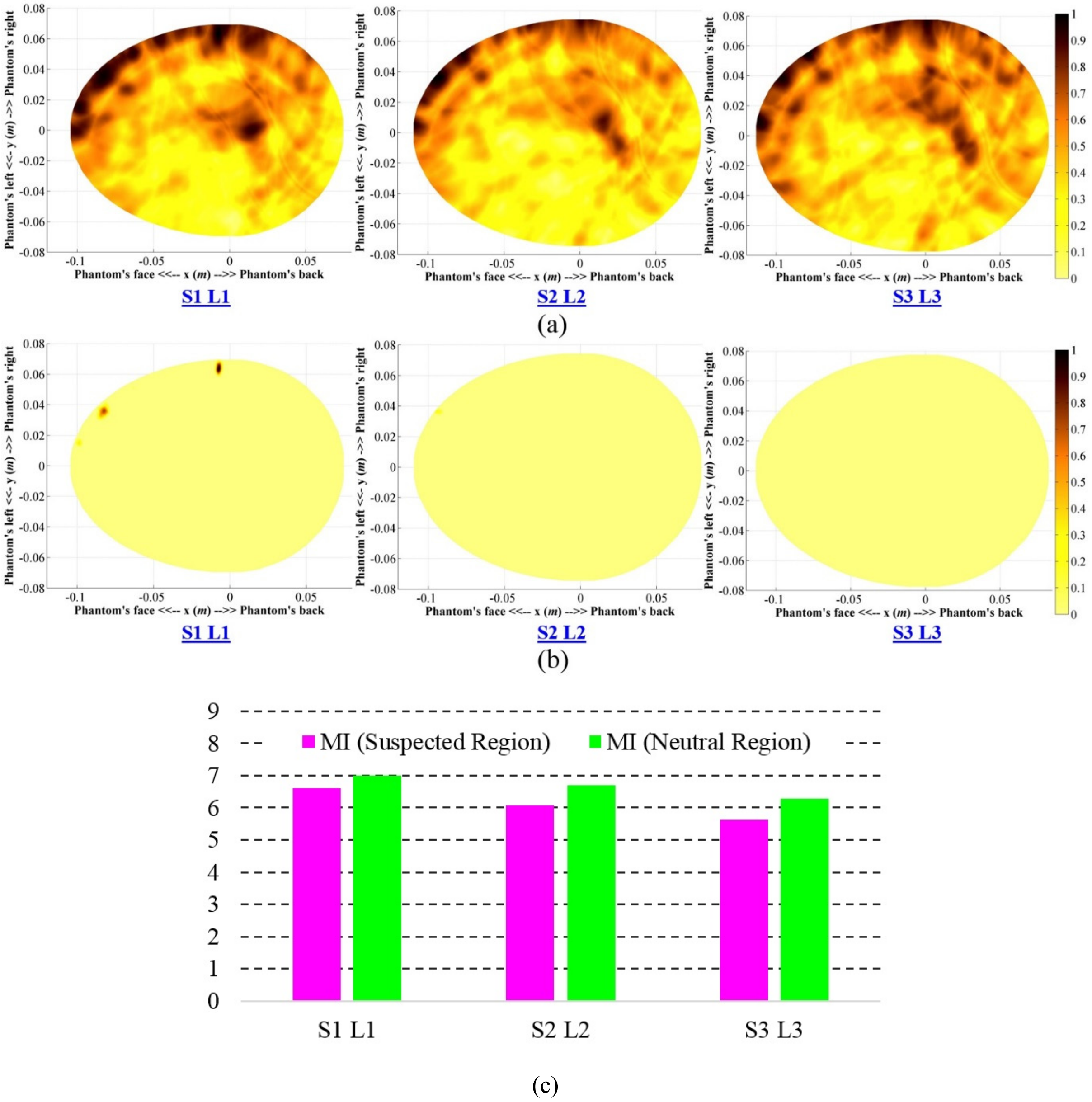

Supplementary Fig. S13. The reconstructed images of three different levels of healthy human volunteer-04 after (a) normalization with respect to individual maximum and minimum of each level, and (b) threshold normalization. (c) The raw maximum intensity values of the suspected and neutral regions at different scanning levels.

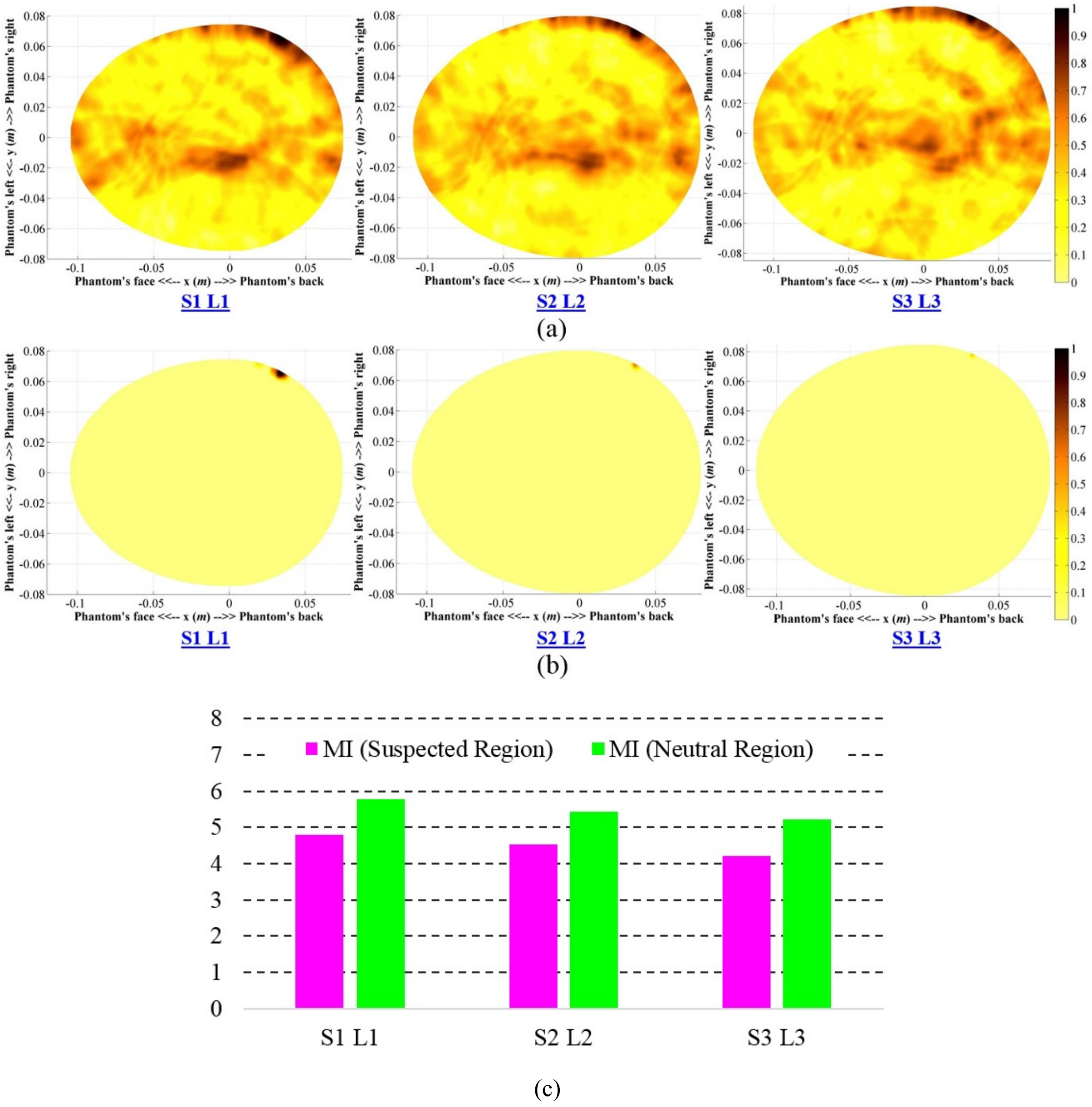

Supplementary Fig. S14. The reconstructed images of three different levels of healthy human volunteer-05 after (a) normalization with respect to individual maximum and minimum of each level, and (b) threshold normalization. (c) The raw maximum intensity values of the suspected and neutral regions at different scanning levels.

Supplementary Table S2: Comparison of data acquisition techniques and quantitative matrices of the reconstructed images among the presented research with the state-of-the-art microwave imaging works

References	Number of data samples around the head	Utilized Phantoms	Quantitative matrices*			Comments		
			Q (dB)	γ (ratio)	δ (mm)	Scanning procedure	Data acquisition time	Localization of target
[33]	32	Realistic head	9.96	1.17	3.54	Manual, involves rotation	4 minutes	2D
[34]	40	Realistic head	10.4	1.25	4.76	Automated, involves rotation	1 minute	2D
This Work	16	Realistic head	12.78	1.59	1.43	Automated, does not require rotation	2 seconds	3D
[36]	32	-	-	-	-	Automated, does not require rotation	4 seconds	2D
[37]	160	Realistic head	-	-	-	Automated, does not require rotation	12.5 seconds	3D
[58]**	200	Simplified, smaller breast	14.1	-	-	Automated, involves rotation	30 minutes	3D
[59, 60]**	60	Simplified, smaller breast	-	16.4 dB (16.2 dB in this work)***	-	Automated, does not require rotation	30 seconds	3D

* The quantitative matrices, average signal to clutter ratio function, Q, maximum signal to clutter ratio, γ and accuracy indicator, δ are respectively calculated following equations (11), (12) and (13). Averages of the quantitative matrices are taken into consideration for reconstructed images with 2×2 cm^2 cross sectional targets in multiple locations are available.

** In order to make a fair comparison and due to the lack of complete microwave imaging prototypes based on similar radar based principles like the current research work, the comparison is done with a breast cancer detection prototype. It is worth to mention that the head imaging poses more challenges in detection owing to higher losses due to high permittivity and conductivity of the head tissues (ref. 62) when compared to breast imaging. Nonetheless, head imaging systems need to potentially image bigger imaging areas (longest and smallest diameters are typically around 20 and 15 cm (see Fig. 6)) in comparison to those of (longest and smallest diameters are maximum 12 and 12 cm (see ref. 58-60)) breast imaging systems. The cancer targets have volumes of 2×1×2 cm^3 and 1×1×1 cm^3 for respectively ref. 58 and 59. Thus, compared to the proposed prototype in some cases the results may seem to be better, but the improvement in case of breast imaging is not conclusive due to their utilized simpler and smaller imaging domain, while it is seen that with smaller head phantoms the imaging performance increases owing to the stronger received scattering signals (ref. 34).

*** The presented value from ref. 59 states the maximum signal to mean clutter ratio for the reconstructed images which is 16.2 dB for the resultant images from the current work.

Supplementary References

[67] Elsherbini, A., Sarabandi, K. ENVELOP antenna: a class of very low profile UWB directive antennas for radar and communication diversity applications. *IEEE Trans. Antennas Propag.* **61(3)**, 1055–1062, (2012).

[68] Yang, J., Kishk, A. A novel low-profile compact directional ultra-wideband antenna: the self-grounded bow-tie antenna. *IEEE Trans. Antennas Propag.* **60(3)**, 1214–1220, (2012).

[69] Mobashsher, A. T., Abbosh, A. CPW-fed low-profile directional antenna operating in low microwave band for wideband medical diagnostic systems. *Electron. Lett.* **50(4)**, 246-248, (2014).

[70] Bukhari, H., Sarabandi, K. Ultra-wideband printed slot antenna with graded index superstrate. *IEEE Trans. Antennas Propag.* **61(10)**, 5278–5282, (2013).

[71] Mobashsher, A. T., Abbosh, A. Slot-loaded folded dipole antenna with wideband and unidirectional performance for L-band applications. *IEEE Antennas Wirel. Propag.* **13**, 798-801, (2014).

[72] Rezaeieh SA, Zamani A, Bialkowski KS, Mahmoud A, Abbosh AM. Feasibility of using wideband microwave system for non-invasive detection and monitoring of pulmonary oedema. *Sci. Rep.* **5**, 14047, (2015). doi:10.1038/srep14047.

[73] Mobashsher, A. T., Abbosh, A. Development of compact directional antenna utilising plane of symmetry for wideband brain stroke detection systems. *Electron. Lett.* **50(12)**, 850-851, (2014).

[74] Mobashsher, A. T., Abbosh, Compact 3-D slot-loaded folded dipole antenna with unidirectional radiation and low impulse distortion for head imaging applications. *IEEE Trans. Antennas Propag.* **64(7)**, 3245-3250, (2016).
